# Boosting in-plane anisotropy by periodic phase engineering in two-dimensional VO_2_ single crystals

**DOI:** 10.1016/j.fmre.2021.11.020

**Published:** 2021-11-26

**Authors:** Meng Ran, Chao Zhao, Xiang Xu, Xiao Kong, Younghee Lee, Wenjun Cui, Zhi-Yi Hu, Alexander Roxas, Zhengtang Luo, Huiqiao Li, Feng Ding, Lin Gan, Tianyou Zhai

**Affiliations:** a State Key Laboratory of Materials Processing and Die and Mould Technology, School of Materials Science and Engineering, Huazhong University of Science and Technology, Wuhan 430074, China; b Centre for Multidimensional Carbon Materials, Institute for Basic Science, School of Materials Science and Engineering, Ulsan National Institute of Science and Technology, Ulsan, South Korea; c Center for Integrated Nanostructure Physics, Institute for Basic Science, Sungkyunkwan University, Suwon, South Korea; d State Key Laboratory of Advanced Technology for Materials Synthesis and Processing, Nanostructure Research Centre, Wuhan University of Technology, Wuhan 430074, China; e Department of Chemical and Biological Engineering, The Hong Kong University of Science and Technology, Clear Water Bay, Kowloon, Hong Kong, China

**Keywords:** Periodic phase engineering, Two-dimensional VO_2_, Interfacial strain, In-plane anisotropy, Electrical anisotropy, Chemical vapor deposition

## Abstract

In-plane anisotropy (IPA) due to asymmetry in lattice structures provides an additional parameter for the precise tuning of characteristic polarization-dependent properties in two-dimensional (2D) materials, but the narrow range within which such method can modulate properties hinders significant development of related devices. Herein we present a novel periodic phase engineering strategy that can remarkably enhance the intrinsic IPA obtainable from minor variations in asymmetric structures. By introducing alternant monoclinic and rutile phases in 2D VO_2_ single crystals through the regulation of interfacial thermal strain, the IPA in electrical conductivity can be reversibly modulated in a range spanning two orders of magnitude, reaching an unprecedented IPA of 113. Such an intriguing local phase engineering in 2D materials can be well depicted and predicted by a theoretical model consisting of phase transformation, thermal expansion, and friction force at the interface, creating a framework applicable to other 2D materials. Ultimately, the considerable adjustability and reversibility of the presented strategy provide opportunities for future polarization-dependent photoelectric and optoelectronic devices.

## Graphical abstract

**Figure:**
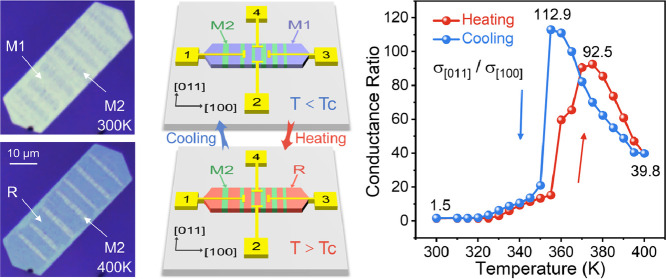

## 1 Introduction

Anisotropy is a widely observed phenomenon in crystalline materials, in which the intrinsic structural asymmetry offers distinct and polarization-dependent responses of optical [1], electrical [2], thermal [3], and magnetic [4] properties. Such structurally tuned materials provide an additional degree of freedom for the modulation of physical and chemical properties. In-plane anisotropy (IPA), first proposed in 2D black phosphorus (BP) [5,6], has increasingly gained traction, with further expansion of its applications to 2D materials. A variety of in-plane polarization-dependent materials have been used in, for example, photodetectors [7,8], synaptic transistor [9], digital inverters [10], and non-volatile memories [11]. The structural asymmetry (e.g., orthorhombic, monoclinic, and triclinic crystal systems), however, elicits a weak intrinsic IPA of about 10^0^ to 10^1^, thus obscuring reliable detection of polarization-dependent signals.

The high anisotropy in materials remains a primary concern and has been attempted to be controlled through local structure modulation [12], [13], [14], [15], alloy/doping [16,17], strain engineering [18], [19], [20], and external field [21], [22], [23]. Among these methods, the main source of anisotropy is still the intrinsic asymmetry of structure, which however is hardly altered and therefore provides restricted enhancement of IPA modulation. This raises questions about whether existing approaches can overcome the limitation and thereby improve the IPA modulation in 2D materials.

Here we demonstrated a novel periodic phase engineering strategy to enhance the IPA in 2D VO_2_ single crystals by introducing alternant monoclinic (M, insulating) and rutile (R, metallic) phases under tunable interfacial thermal strain. 2D VO_2_ single-crystalline nanoflakes were grown on the mica substrate by chemical vapor deposition (CVD), in which two alternating monoclinic phases, M_1_ and M_2_, were formed in VO_2_ single crystals by the interfacial thermal strain on the mica substrate. This alternant M_1_/M_2_ pattern can further reversibly evolve into the R/M_2_ pattern by modulating the interfacial thermal strain, which can be precisely depicted and predicted by a general theoretical model. On this basis, we demonstrated in VO_2_ nanoflakes a striking modulation of electrical IPA over a wide range that spans two orders of magnitude, reaching an unprecedented IPA of 113. This periodic phase engineering therefore gains new insight on the full potential of IPA for future applications.

## 2 Material and methods

### 2.1 Synthesis of VO_2_ nanoflakes

VO_2_ nanoflakes were synthesized by the chemical vapor deposition method, in which 15 mg V_2_O_5_ powder was mixed with 5 mg NaCl powder to accelerate evaporation and was used as the source altogether. Fluorophlogopite mica KMg_3_(AlSi_3_O_10_)F_2_ was used as the substrate in the deposition at 780 °C under the protection of 50 sccm high-purity argon. After about 30 minutes of deposition, VO_2_ nanoflakes were observed to have grown on the mica substrate.

### 2.2 Transfer of VO_2_ nanoflakes

The mica substrate with samples on its surface was first covered by a thin layer of PMMA (poly(methyl methacrylate)) through spin coating (4000 rpm, 60s) and then heated on a hot plate at 150 °C for 5 minutes. A thin layer of PPC (Poly (propylene carbonate), v.15% in v. 85% anisole) was subsequently coated over the PMMA coating and heated at 95 °C for another 5 minutes. Finally, the whole substrate was submerged in DI water for 30 minutes before the VO_2_ samples were embedded in the polymer coating layer and exfoliated from the mica substrate. All nanodevices in this study were directly fabricated on the mica substrate without transfer operation to retain the phase pattern in the VO_2_ nanoflakes.

### 2.3 Characterization and simulation of VO_2_ nanoflakes

Optical images of the sample were taken by Olympus optical microscopy (BX51). Raman spectra were collected by WITec confocal Raman system (Alphas 300 RAS) under a 532 nm laser. A laser power density of 0.5 mW was used for usual tests, but the power was increased for phase transition tests. Varied-temperature Raman measurement was conducted in an Oxford cryostat (Microstat HiRes 2). All nanodevices were fabricated by the E-beam lithography system (FEI Quanta 650 SEM, equipped with the Raith Elphy Plus pattern processor) and measured in a Lakeshore cryogenic probe station (CRX-6.5K) with a Keithley semiconductor parameter analyzer (B1500A). The wrinkles in VO_2_ nanoflake were simulated by a 3D finite element model using the commercial software ABAQUS. Related parameters extracted from experimental results can be found in the theoretical section of the Supporting Information.

## 3 Results and discussions

### 3.1 Identification of phases in VO_2_ nanoflakes

**Fig. 1:**
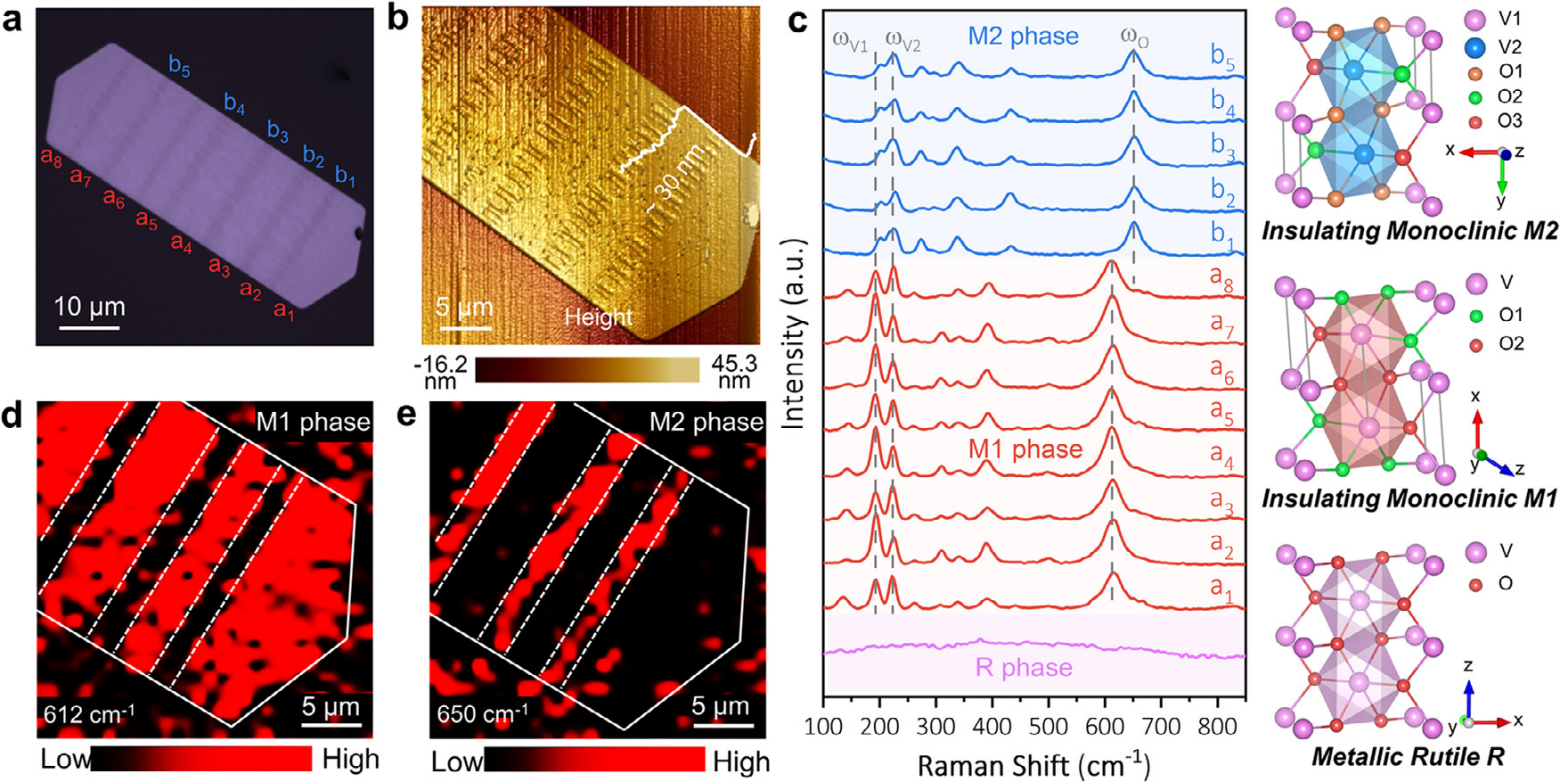
**Optical characterization of thin VO**_**2**_**nanoflakes with M**_**1**_**and M**_**2**_**phase.** (a) Optical microscope image of as-synthesized VO_2_ thin nanoflakes on the mica substrate. (b) AFM image and the corresponding height profile. (c) Raman spectra acquired from different positions in (a) and crystal structures of R, M_1_, and M_2_ phases. (d, e) Raman mapping images of 612 cm^−1^ (M_1_) and 650 cm^−1^ (M_2_) vibrational modes in (a), respectively. The Raman spectra and mapping were measured by a 633 nm laser at 0.5 mW.

VO_2_ nanoflakes were grown on a fluorophlogopite mica substrate by the CVD method and developed an obvious piano keyboard-like alternating pattern of bright and dark stripes, which were perpendicular to the long axis of the sample at room temperature (Fig. S1). This intriguing phenomenon has not been observed in previous works since similar patterns in strained VO_2_ nanowires were reported only at elevated temperatures [24,25]. A typical VO_2_ nanoflake shown in Fig. 1a had a thickness of about 30 nm as measured by an atomic force microscope (AFM, Fig. 1b). Interestingly, periodic wrinkle arrays formed in the dark stripes, but both the stripes and the wrinkles disappeared after sample transfer or did not form in thick samples (Fig. S1-S3). Since this feature was caused by the interfacial stress between VO_2_ and the mica substrate, the stress would definitely dissipate after sample exfoliation. On the other hand, wrinkles failed to form in thick samples because the required bending energy exceeded the strain energy that served as the driving force of wrinkle formation (See theoretical section of this paper for further discussion). The Raman spectrum of each bright (a) and dark (b) stripe that developed on the sample detected two types of the monoclinic phase, M_1_ and M_2_, respectively (Table S**1**). In contrast, a uniform Raman signal from the M_1_ phase alone was detected from the thick sample (Fig. S4). To further investigate the observed phenomenon, the Raman spectra of the M_1_, M_2_, and R phases were aligned and labeled with the corresponding unit cells (Fig. 1c). First, the R phase belongs to the *p4_2_/mnm* (#136) space group, where each V^4+^ ion is surrounded by six O^2−^ ions to form a slightly distorted octahedral VO_6_ unit with uniform V-V bond lengths [26]. As mentioned above, the R phase is metallic and thus shows no obvious signal of Raman scattering [27]. Second, the M_1_ phase is the most reported insulating phase and belongs to the *p2_1_/c* (#14) space group. The V-V bonds in the M_1_ phase have two unequal lengths as the dimerization of the V atoms leads to a slight deviation from the **c** axis [26]. Although the Raman spectrum of the M_1_ phase contains many peaks, we focused only on the three strongest peaks at ∼ 192 (ɷ_v1_), 224 (ɷ_v2_) and 612 cm^−1^ (ɷ_o_) [28]. Third, the M_2_ phase, which belongs to the *C2/m* (#12) space group, may emerge from either the R or the M1 phase under tensile stress along [001]_R_ or [100]_M1_ [29] (**b_M2_**//**a_M1_**//**c_R_**). Like the M_1_ phase, there are two V-V bond types in the M_2_ phase, but the dimerization of V occurs directly to the **c** axis without deviation. While the Raman spectrum of the M_2_ phase shares similar peaks with the M_1_ phase, a tiny blue shift at the vibrational modes of ɷ_v1_ and ɷ_v2_ and a large blue shift (from 612 to 650 cm^−1^) at the vibrational mode of ɷ_o_ were both noticed [29]. The reliability of identifying the M_2_ phase from the Raman spectrum was also verified from the bent VO_2_ sample (Fig. S5), in which the M_2_ phase formed under tension [30,31]. To verify the distribution of the M_1_/M_2_ phase in VO_2_, a mapping of the sample was performed using peaks 612 cm^−1^ and 650 cm^−1^ (Fig. 1d,e), showing that the alternating pattern exactly matched the optical image in Fig.1a.

### 3.2 Periodic phase engineering in VO_2_ nanoflakes

**Fig. 2:**
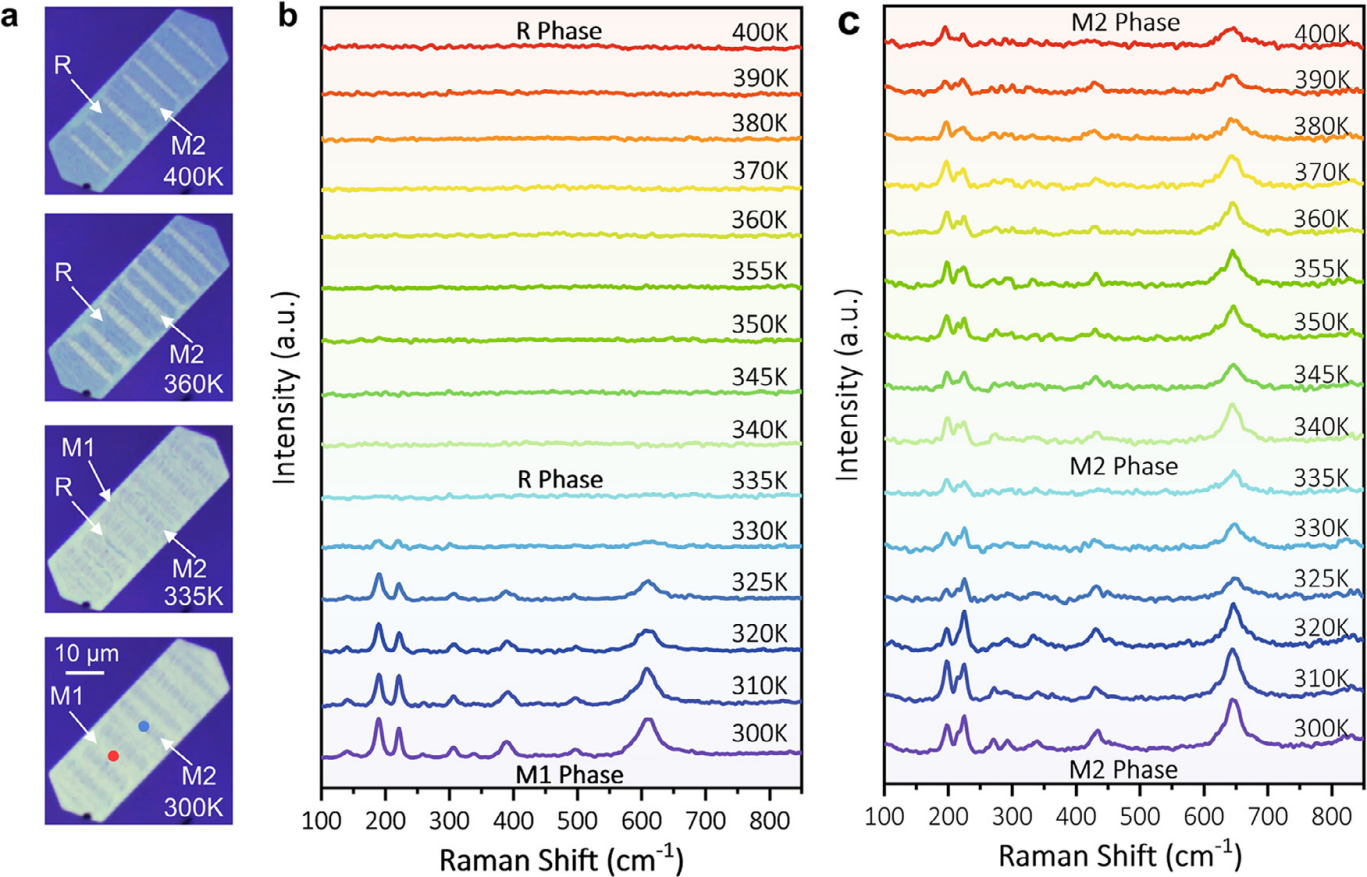
**Varied-temperature Raman of the thin VO**_**2**_**nanoflake on mica.** (a) Optical microscope images of the thin VO_2_ nanoflake under different temperatures. (b, c) Raman spectra in-situ acquired from the bright (M_1_ phase, red point) and the dark (M_2_ phase, blue point) stripes in (a) under different temperatures.

Metal-insulator transition in VO_2_ could be easily triggered by thermal treatment, but how the M_1_/M_2_ pattern evolves with thermal treatment is an intriguing subject for study. Here we in-situ monitored the evolution of the Raman spectra of the M_1_ and the M_2_ phases between 300 K and 400 K. As shown in the optical images in Fig. 2a, the M_1_/M_2_ pattern in the 30-nm VO_2_ nanoflake displayed a reversed brightness contrast as the sample was heated up from 300 to 400 K (more images in Fig. S6), that is, the M_2_ stripes changed from dark to bright and the M_1_-stripes from bright to dark. Combined with the Raman spectra in Fig. 2b,c, the M_1_ stripes exhibited a significant decrease of Raman intensity from 325 K and completely transformed into the R phase (without Raman signal) between 335 and 340 K (See infrared reflection mapping in Fig. S7), concurring with the bulk result [32]. On the contrary, the Raman signal of the M_2_ phase was consistent throughout the temperature range of study, except for the attenuation of intensity at higher temperatures, which could be attributed to the shrinking of the M_2_ phase or the temperature effect on Raman [33]. In this case, it is worth noting that the orientation and location of the R phase conversion were restricted within the M_1_ stripes throughout the temperature range, except at higher temperatures in which the M_2_ stripes also developed into the R phase. For comparison, we performed the same operation on a thick sample (>100 nm). The R phase, consistent with the trend of the M_1_ stripes, emerged beginning from 325 K although randomly over the sample area and then covered the whole sample at 360 K (Fig. S8). Cooling both samples illustrated the reversibility of phase transition albeit again in a disordered manner in the thick sample. On the other hand, regardless of heating or cooling, the phase in the thin sample exhibited a high consistency and thus a favorable predictability in terms of orientation and location (Fig. S9). This fascinating real-time phase transition process was captured in Video S1. As for the thin sample exfoliated from the mica substrate, the phase transition behavior was similar to that of the thick sample (Fig. S10), suggesting the key role of interfacial stress. Laser could also trigger a phase transition behavior identical to the effect of heat as demonstrated by both (a) the transformation of the M_1_ stripes to the R phase starting from a laser power intensity of 1.5 mW (633 nm) and (b) the resistance against phase transition of the M_2_ stripes until a power intensity of 2.5 mW (Fig. S11)

### 3.3 Theoretical model for periodic phase engineering

**Fig. 3:**
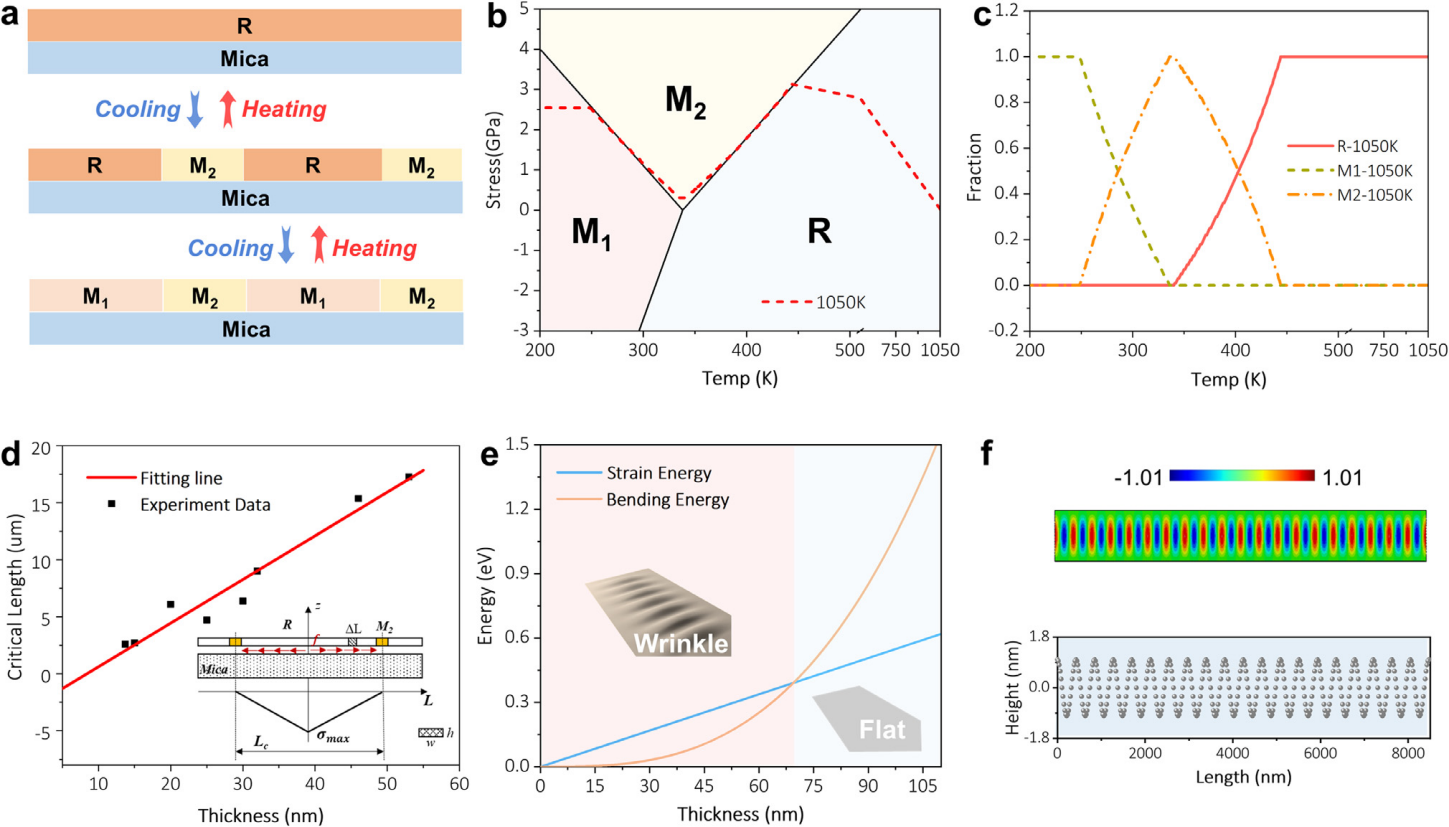
**Theoretical study of the stripes and the wrinkles.** (a) Schematic diagram of the VO_2_ phase transition during the heating and cooling process. (b, c) Stress distribution and proportion of R, M_2_, and M_1_ phases evolving with temperature, respectively. (d) The relationship between the periods of the pattern of the stripes and the thickness of the VO_2_ nanoflakes (The inset denotes the analytical model for the pattern study). (e) The critical thickness estimation for wrinkle formation in the M_2_ phase of the VO_2_ nanosheet. (f) FEM results of the surface topography (upper) and the wrinkle height profile (nether) of the M_2_ phase in the VO_2_ nanosheet.

Both analytical model and finite element method (FEM) simulation were employed to unveil the formation mechanism of the stripes and the wrinkles. Here we carried out the simulation only on the cooling process (1050 to 300 K) because the heating process is an equivalent but reversed process as elaborated above. The phase transition during the cooling process, which followed the simple model shown in Fig. 3a, can be separated into two stages according to our experimental data, the R-M_2_ transition (from 440 to 340 K) and the R-M_1_/M_2_-M_1_ transition (from 340 to 300 K). The proposed theoretical model to explain the R-M_2_-M_1_ phase transition (Fig. S12) is expressed as:

(1) $$E_{T}=\alpha_{1}E_{R}\left(T\right)+\alpha_{2}E_{M_{1}}\left(T\right)+\left(1-\alpha_{1}-\alpha_{2}\right)E_{M_{2}}\left(T\right)$$

where the three terms are the total energies of the R, M_1_, and M_2_ phases, respectively, and comprise the bulk free energy and the elastic strain energy. The proportion of the R and M_1_ phase, $\alpha_{1}\;$and $\alpha_{2}$, could be calculated by minimizing the total energy or $\frac{\partial E_{T}}{\partial \alpha_{1}}=0$ and $\frac{\partial E_{T}}{\partial \alpha_{2}}=0$ (Supplementary Section 11.1). Fig. 3b shows the phase diagram [31] and the stress distribution of VO_2_ evolving with temperature. When lowering the temperature, the stress of the VO_2_ nanoflake increases first and then decreases along the R-M_2_ phase transition boundary, indicating the R-M_2_ phase transition process. Subsequently, the stress increases along the M_2_-M_1_ boundary, implying the M_2_-M_1_ phase transition process, before ultimately reaching the M_1_ phase. Meanwhile, the proportion of the R, M_2_, and M_1_ phases evolving with temperature is shown in Fig. 3c. As temperature cools down to about 445 K, the M_2_ phase gradually appears and grows to its maximum at about 338 K with the decrease of the R phase. Further lowering of the temperature results in the shrinking and the growth of the M_2_ and the M_1_ phases, respectively, agreeing with the presented experimental observation. Fig. 3d shows the periods of the pattern of stripes (data collected from Fig. S13), which expands almost linearly with increasing thickness of the VO_2_ nanoflakes. These periods were modeled (the inset in Fig. 3d) as a function of the thickness of the VO_2_ nanoflakes and the frictional shear stress between the nanoflakes and the mica substrate (Supplementary Section 11.2.1). According to this model, the frictional shear stress was roughly estimated to be 8.1 MPa, which is on the same order of magnitude as that of ZnO-mica interface (5.1 MPa) [34], a member of Van der Waals oxide heteroepitaxy family [35]. Furthermore, we found that the wrinkles disappeared when the thickness of VO_2_ nanoflakes is greater than 66 nm (Fig. S13). To understand the formation of wrinkles, the profile of the wrinkles, which were described with an average wrinkle height of 1.5 to 2.0 nm and a wavelength of ∼ 380 nm through AFM, was depicted in an analytical model (Fig. S14-15). To form a wrinkle, the compressive strain energy should be greater than the bending energy shown in Fig. 3e, which shows that the increase of the former and the latter with growing thickness of VO_2_ nanoflakes are linear and cubic, respectively. Thus, the wrinkles can only be theoretically formed below approximately 70 nm in thickness (Supplementary Section 11.2.2), which is consistent with our experimental data. To investigate the wrinkle in depth, we further built a 3D finite element model to simulate the wrinkles in thin VO_2_ samples via buckling [36] and post-buckling analysis [37] (Fig. S16). We found that when the wrinkle height is between 1.5 and 2.0 nm, the simulated wavelength of the wrinkles (Fig. 3f) matches well with the results of our work.

### 3.4 Effect of periodic phase engineering on IPA of VO_2_ nanoflake

**Fig. 4:**
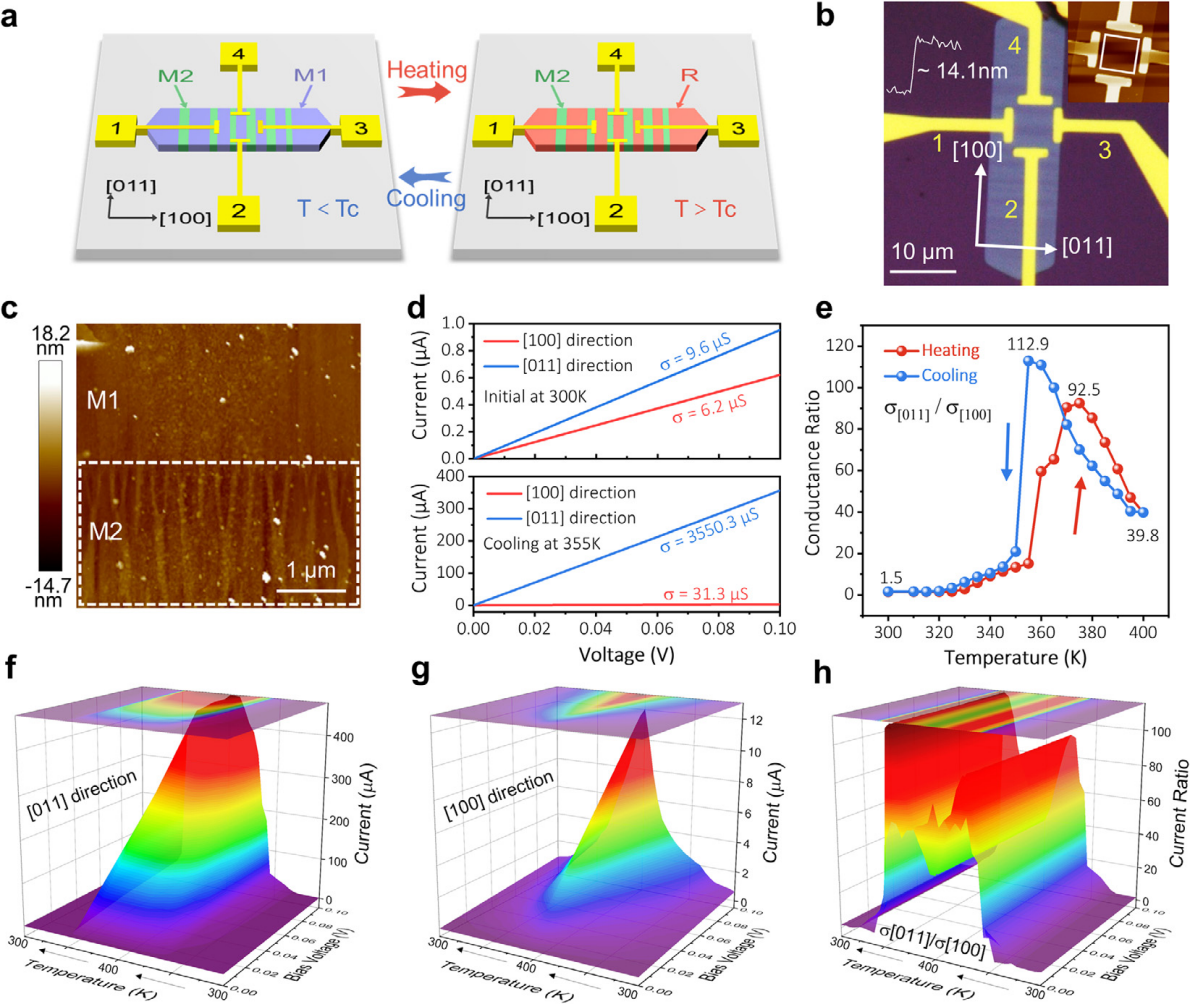
**Modulation of electrical anisotropy of the striped VO**_**2**_**device.** (a) Design of the in-plane electrical anisotropy measurement by cross-type electrode pairs. (b) Optical image of the striped VO_2_ device and its AFM image (inset). (c) Enlarged AFM image of the solid boxed area in (b). (d) The initial current–voltage curves along the [011] and the [100] directions at 300 K and the maximum current difference curves along the [011] axis and the [100] axis during the cooling process at 355 K. (e) The evolution of the conductance ratio in a cycle covering the temperature range from 300 to 400 K. (f, g) 3D images of the relationship between the working current and the bias voltage along [011] and [100], respectively, in the temperature cycle. (h) The corresponding conductance ratio of [011]/[100] in the temperature cycle. All measurements are conducted in the ambient environment.

To verify the modulation effect on the IPA, devices with cross-type electrode pairs were fabricated onto thin VO_2_ nanoflakes. As illustrated in Fig. 4a, electrode pairs 1-3 and 2-4 were deposited along the longer axis ([100]_M1_) and the shorter axis ([011]_M1_) of the VO_2_ nanoflake, respectively, wherein each pair dominated the same channel length and width. In this design, the insulating M_1_ stripes turned into metallic R stripes upon heating, while the insulating M_2_ stripes remained unchanged, causing disparity in the electrical conductivities of [100]_M1_ (with M_2_/R interfaces) and [011]_M1_ (shorted by metallic R phase). It should be emphasized here that at least one phase interface (M_1_/M_2_) must be included in each channel. An optical image of the device is shown in Fig. 4b, with the corresponding AFM image (inset) indicating the VO_2_ to be about 14 nm. Enlarged AFM images (Fig. 4c) confirmed the flat surface in the M_1_ stripes and wrinkles in the M_2_ stripes as expected. The initial IPA ratio, defined as the conductance ratio of [011] to [100], was initially about 1.5 at 300 K and achieved its maximum value of about 112.9 at 355 K, during which the M_1_ stripes have already transitioned completely to the R phase. Moreover, at this point the converted M_2_ stripes were still too small to reduce the IPA ratio significantly (Fig. 4d; another device with a similar trend is shown in Fig. S17 & Table S2). A complete cycle of conductance ratio evolution in the range from 300 to 400 K is summarized in Fig. 4e, wherein an apparent hysteresis is observed between the heating and cooling curves. Such a thermal hysteresis is a typical character of the phase transition in VO_2_, resulting from the lattice incompatibility between the transformed and the parent phases [38]. Importantly, the excellent reversible and strict phase transition defined by the interfacial strain is reproducible with IPA modulation (Supplementary Fig. S18). We further compared the IPA ratios with other common anisotropic 2D materials and enhanced strategies (Table S3). To examine the evolution of the conductance along the different axes, the correlations between the working current and the bias voltage in the temperature cycle along [100] and [011] were individually plotted in 3D mode. The conductance along [011] abruptly changed at an almost fixed temperature (Fig. 4f), whereas the corresponding bias voltage along [100] dropped with rising temperature (Fig. 4g). Such nonsynchronous change demonstrated the ratio reached its maximum at an optimal temperature (Fig. 4h). A more detailed model that describes this electrical transport evolution due to phase transition can be found in Fig. S19-S22. The conductance curves in the thick VO_2_ nanoflake displayed a steep slope near the temperature of phase transition (Supplementary Fig. S23), agreeing with a previous report that attributed the absence of a sudden change in value to the existence of stress in VO_2_ [39]. It must be emphasized that this demonstration of property modulation is likewise applicable in other kinds of properties. For example, the tunability of optical and thermal conductivities through this strategy can be achieved, considering the diversity of features realized here between monoclinic and rutile VO_2_.

## 4 Conclusion

In summary, we demonstrated a novel periodic phase engineering strategy to elevate the small IPA in 2D structures by introducing alternant phases. This technique enabled the modulation of IPA without depending on structural asymmetry alone but by phase type and their spatial distribution as well. On this basis, we achieved a remarkable improvement of the electrical IPA in VO_2_ nanoflakes by two orders of magnitude and built a general theoretical model to accurately depict and predict this intriguing phase evolution in 2D materials. The full potential of this strategy, however, cannot be entirely understood if considering the 10^3^ to 10^5^ times resistivity difference [40] between the metallic and the insulating phases of VO_2_. It is also worth pointing out that the interfacial interaction is largely attributed to the strain caused by the mismatch of thermal expansion coefficients at the interface, highlighting the importance of selecting a proper substrate. But such method of inducing phase transition may be insufficient and thus ineffective for phase modulation in other 2D materials, like TMDs. Other ways to strengthen interfacial interaction may be further explored, including piezoelectric substrates for larger interfacial strain [41], surface morphology design for enhanced local strain [42], and tunable friction force [43]. The construction of a global energy background, such as temperature or charge doping, would also facilitate the phase transition with higher activation energy requirements.

## Declaration of Competing Interest

The authors declare that they have no conflicts of interest in this work.

## Appendix Supplementary materials

Image, application 1

Image, video 2

Image, video 3
