# Supplementary material for: Boosting in-plane anisotropy by periodic phase engineering in two-dimensional VO2 single crystals
Source: Fundam Res. 2021 Nov 26;2(3):456–61. doi: 10.1016/j.fmre.2021.11.020 (PMC11197522; doi:10.1016/j.fmre.2021.11.020)
Supplement: Supplementary file 1 [file mmc1.docx]

**Supplementary Materials for Boosting in-plane Anisotropy by Periodic Phase Engineering in Two-dimensional VO_2_ Single Crystals**

Meng Ran^a,1^, Chao Zhao^b,1^, Xiang Xu^a,1^, Xiao Kong^b^, Younghee Lee^c^, Wenjun Cui^d^, Zhi-Yi Hu^d^, Alexander Roxas^e^, Zhengtang Luo^e^, Huiqiao Li^a^, Feng Ding^b,^*, Lin Gan^a,^*, Tianyou Zhai^a,^*

^a^*State Key Laboratory of Materials Processing and Die and Mould Technology, School of Materials Science and Engineering, Huazhong University of Science and Technology, Wuhan, 430074 P. R. China*

^b^*Centre for Multidimensional Carbon Materials, Institute for Basic Science, School of Materials Science and Engineering, Ulsan National Institute of Science and Technology, Ulsan, South Korea*

^c^*Center for Integrated Nanostructure Physics, Institute for Basic Science (IBS), Sungkyunkwan University, Suwon, Korea*

^d^*State Key Laboratory of Advanced Technology for Materials Synthesis and Processing, Nanostructure Research Centre, Wuhan University of Technology, Wuhan, 430074 P. R. China*

^e^*Department of Chemical and Biological Engineering, The Hong Kong University of Science and Technology, Clear Water Bay, Kowloon, Hong Kong*

^1^*These authors contributed equally to this work*

1. **Chemical vapor deposition of VO_2_ nanoflakes and identification of crystal phase**

As shown in **Fig. S1a**, VO_2_ nanoflakes were synthesized by the chemical vapor deposition method, in which 15 mg V_2_O_5_ powder was mixed with 5 mg NaCl powder for accelerating evaporation and was used as the source. The deposition used fluorophlogopite mica KMg_3_(AlSi_3_O_10_)F_2_ as the substrate and was conducted at 780 ^o^C under the protection of 50 sccm high-purity gas Ar. After around 30 minutes of growth, VO_2_ nanoflakes grew on the mica substrate as shown in **Fig. S1b**. The various colors indicated the different values of thickness of VO_2_ with observable differences in distinct morphology differences. For instance, thick samples showed uniform surface (**Fig. S1c**), but periodical ripples can be seen in thin samples (**Fig. S1d**). These ripples were induced by the interfacial stress existing between VO_2_ and the mica substrate due to the different thermal expansion coefficients as demonstrated by a schematic drawing in Fig. S1a. XRD data verified VO_2_ nanoflakes to be monoclinic phase based on the sample transferred on SiO_2_/Si substrate (**Fig. S1e**). **Fig. S1f-1h** are the HRTEM images of VO_2_ nanoflakes with their clear atomic structure and the SAED patterns, in which the (011) and ($\bar{2}01$) planes of monoclinic VO_2_  may be identified. The uniform distribution of V and O elements in the sample was also acquired by elemental mapping (**Fig. S1i-1j)**. Only the M_1_ phase could be found by TEM even for thin VO_2_ nanoflakes possibly because of the release of interfacial stress during the transfer process. Similarly, only the M_1_ phase could be detected in the XRD spectra acquired from the samples transferred on SiO_2_/Si substrate. Even for the XRD data of the samples grown on the mica substrate, it was also impossible to distinguish the M_2_ phase from the M_1_ phase because their crystal lattice was so close that corresponding XRD peaks overlapped.

**
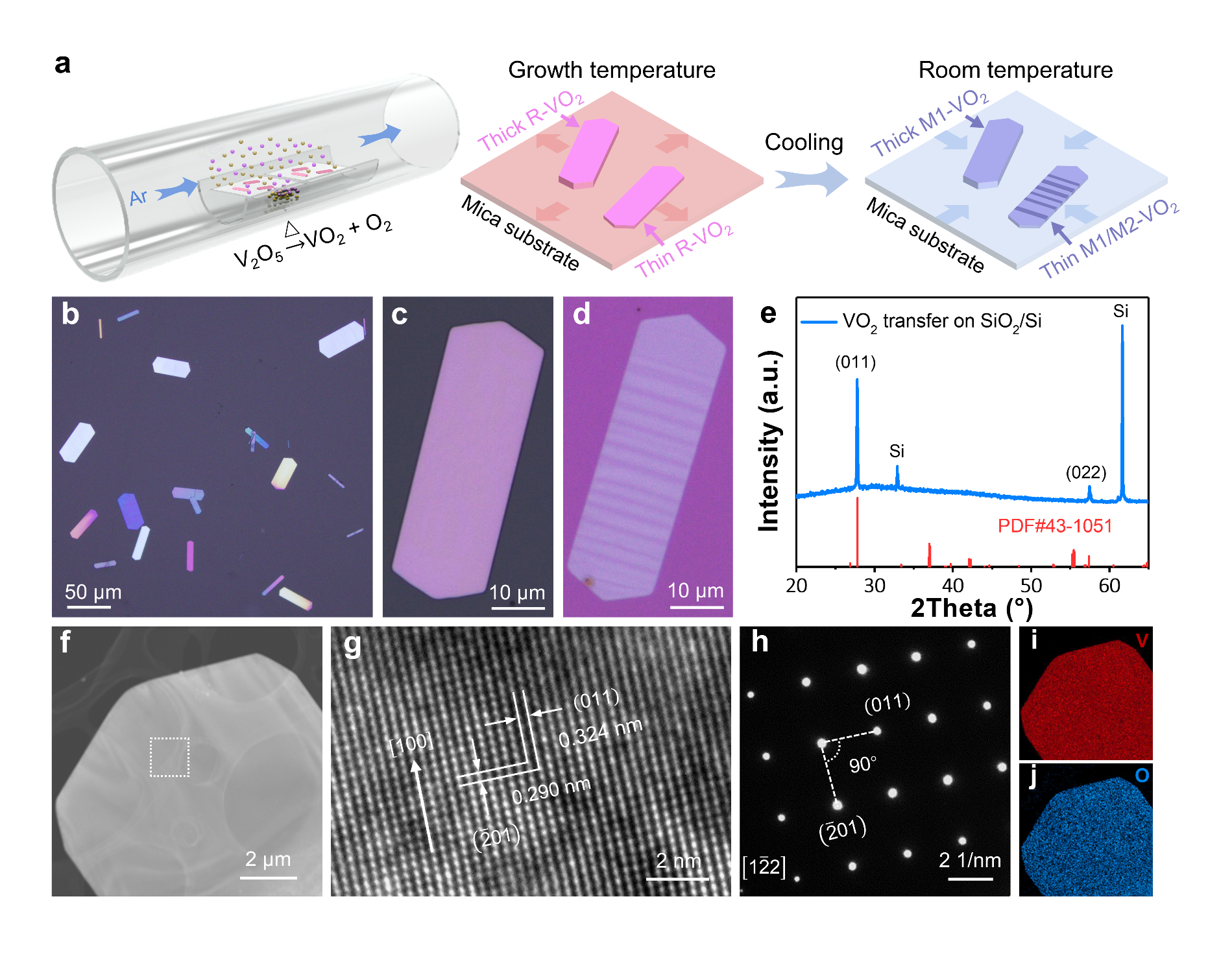
**

**Figure S1. Synthesis and characterization of VO_2_ nanoflakes.** a) Schematic view of the VO_2_ nanoflakes growth process. b) Optical image of as-synthesized VO_2_ nanoflakes on a mica substrate. c, d) Typical optical images of thick and thin VO_2_ nanoflakes on mica, respectively. e) XRD pattern of VO_2_ nanoflakes transferred on SiO_2_/Si substrate. f) Low-magnification image of a VO_2_ nanoflake transferred on a copper grid. g) HRTEM image of the dotted square in (f) and h) the corresponding SAED pattern indexed for monoclinic VO_2_ on the [1-22] zone axis. i, j) V and O elemental mappings of the sample shown in (f).

1. **Comparison of the morphologies of thin and thick VO_2_ nanoflakes**

Clear wrinkles were observed on a thin VO_2_ nanoflake on a mica substrate, whereas a slight potential difference of ~ 12 mV between the bright and the dark stripes was measured from KPFM (**Fig. S2a-b**). In contrast, the surface of a thick VO_2_ nanoflake showed uniform roughness and potential distribution (**Fig. S2c-d**). All stripes and wrinkles disappeared after exfoliating the sample from the mica substrate (**Fig. S2e**) because of the release of interfacial stress.

**
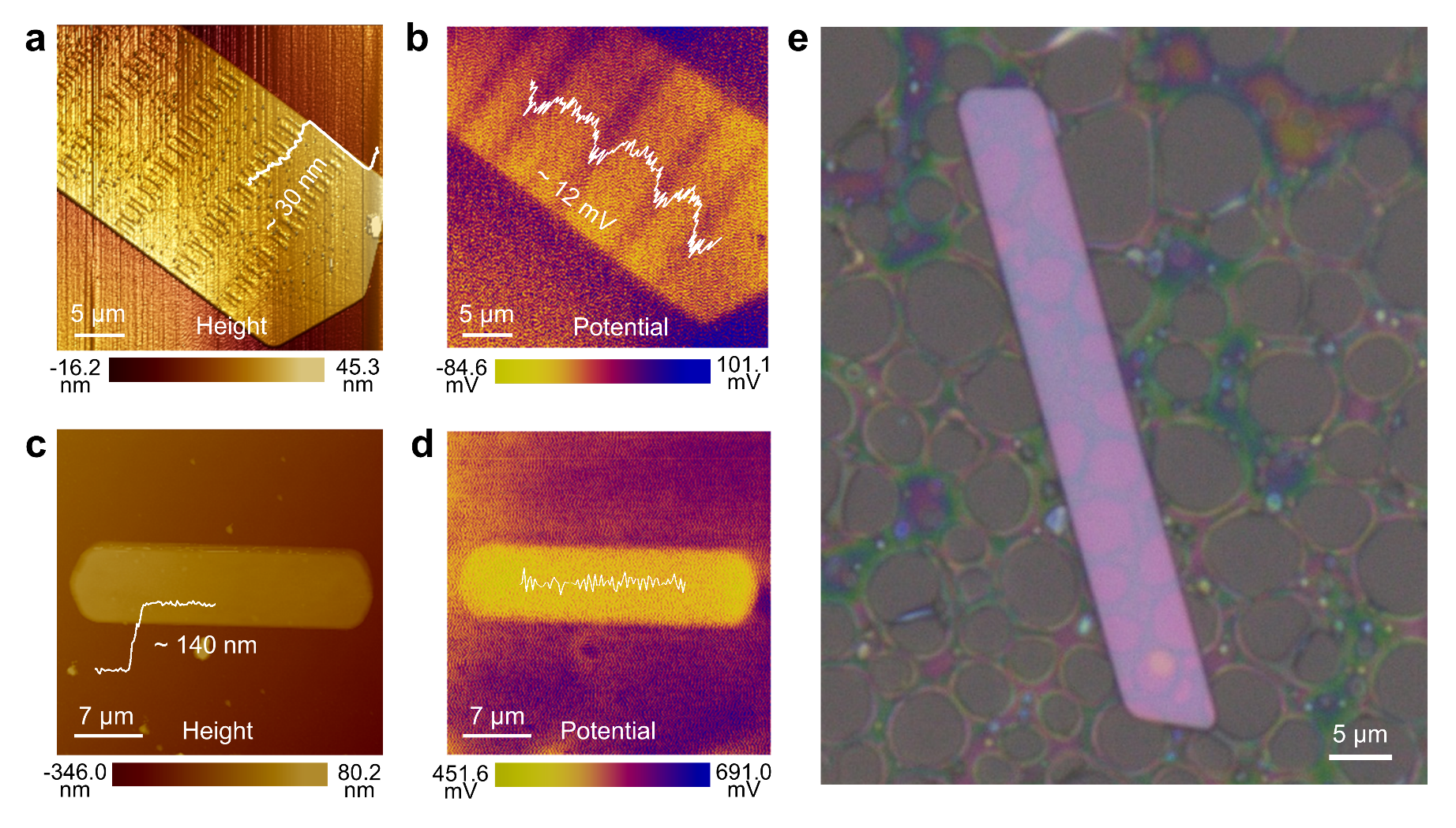
**

**Figure S2. Comparison of the morphologies of VO_2_ nanoflakes with different thicknesses.** a) 3D AFM image and the corresponding height profile of the same thin VO_2_ nanoflake in Fig. 1a. b) KPFM image and the corresponding potential profile along the VO_2_ stripes in Fig. 1a, showing a potential difference of ∼12 mV between bright and dark stripes. c) AFM image and the corresponding height profile of a thick VO_2_ nanoflake. d) KPFM image and the corresponding potential profile on the surface of the thick VO_2_. e) Optical image of the thin VO_2_ nanoflake transferred on a copper grid having a scale bar of 5 µm.

1. **PPC/PMMA-assisted transfer method**

The VO_2_ nanoflakes were transferred via a dry method assisted by PPC/PMMA film to avoid sample etching during the transfer process:

1. All mica substrates with grown VO_2_ nanoflakes were covered with PMMA solution on the surface (4000 rpm, 60 s) and heated at 150^o^C for 5 minutes.

2. The PMMA-coated mica substrates were covered with PPC solution (15% PPC in 85% anisole) on the surface (2000 rpm, 60 s) and heated at 95^o^C for 5 minutes.

3. The PPC/PMMA-coated mica substrates were submerged into de-ionized water for at least 30 minutes, during which each film of PPC/PMMA/VO_2_ was exfoliated by bubbles.

4. The film was fully exfoliated gently using tweezers from a corner of the film and transferred to the target PEN substrate.

5. Each PEN substrate was heated at 60^o^C for 5 minutes to expel air at the interface.

6. The PEN substrates were submerged into hot acetone at about 40 ^o^C until the complete dissolution of the PPC/PMMA film and were rinsed with de-ionized water before drying with Ar gun blowing.

7. Strain was applied on PEN to modulate the stress in VO_2_.

With this method, VO_2_ samples can be transferred to an arbitrary substrate, such as PEN, Copper grid and SiO_2_/Si.


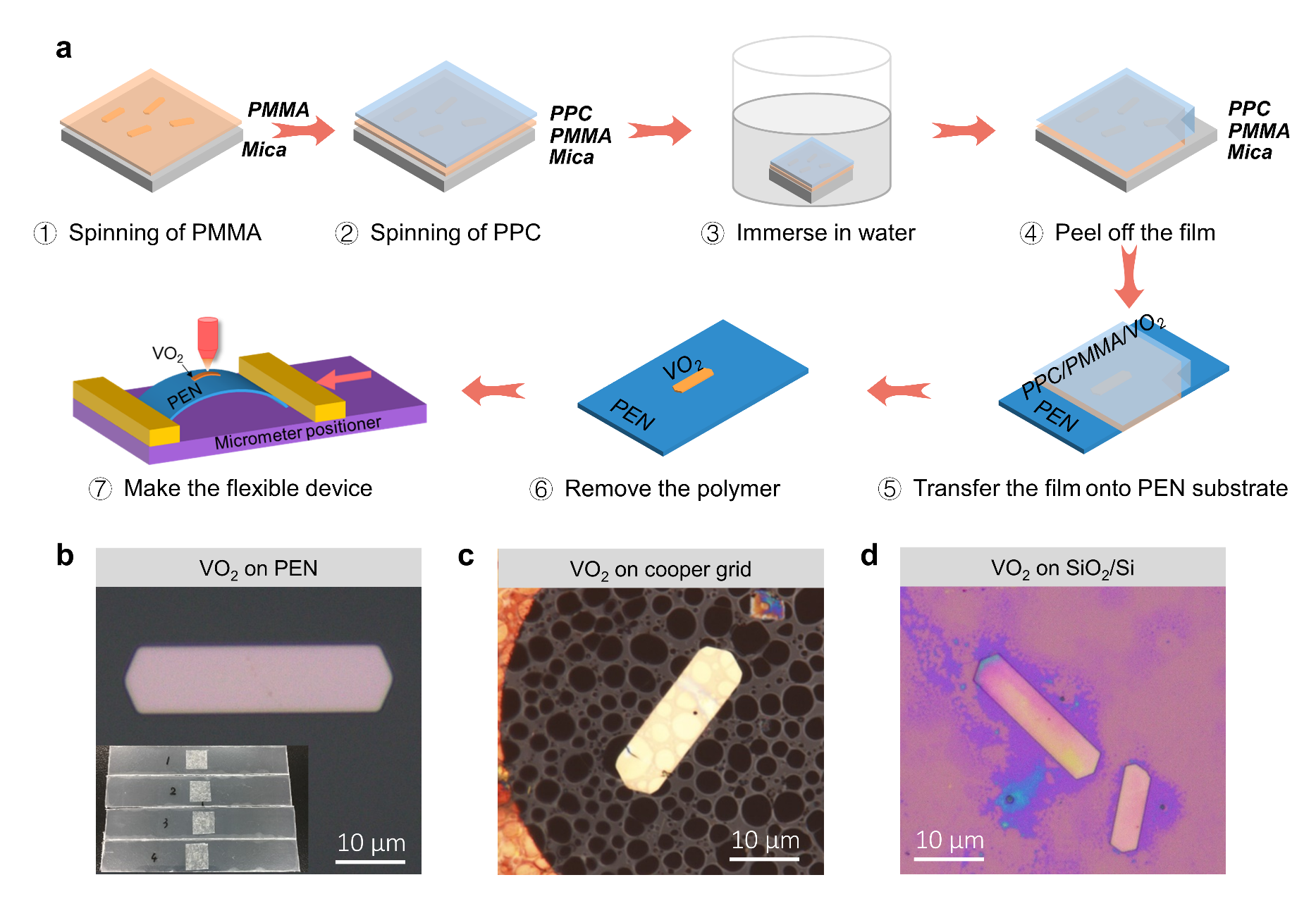


**Figure S3. Transfer process of as-synthesized VO_2_ nanoflakes for following test.** a) PPC/PMMA-assisted transfer process for strain test on the flexible substrate. b-d) Optical images of samples transferred on various substrates.

**Table S1.** Comparison of Raman peaks position between this work and reported works.

| M_1_-VO_2_ | | | M_2_-VO_2_ | | |
| --- | --- | --- | --- | --- | --- |
| Film[^1^](#_ENREF_1) | Nanobeam[^2^](#_ENREF_2) | This work | Film[^1^](#_ENREF_1) | Nanobeam[^2^](#_ENREF_2) | This work |
| 132 | - | 144 | 202 | 199 | 201 |
| 195 | 190 | 192 | 222 | 226 | 225 |
| 224 | 222 | 224 | 275 | 272 | 273 |
| 261 | - | 261 | 297 | 293 | 296 |
| 310 | 306 | 310 | 340 | 338 | 341 |
| 336 | 337 | 340 | 434 | 432 | 433 |
| 386 | 388 | - | 449 | 499 | 500 |
| 391 | - | 391 | 648 | 645 | 650 |
| 439 | - | 440 | 815 | - | 831 |
| 443 | - | - |  |  |  |
| 498 | 496 | 498 |  |  |  |
| 616 | 608 | 612 |  |  |  |
| 831 | - | 824 |  |  |  |

1. **Identification of the phase of thick VO_2_ via Raman spectrum**

A thick VO_2_ nanoflake had a typical Raman spectrum of the M_1_ phase[^1^](#_ENREF_1). The surface of the sample was rather uniformly reflected from the mapping image under a fingerprint peak of 612 cm^-1^.

**
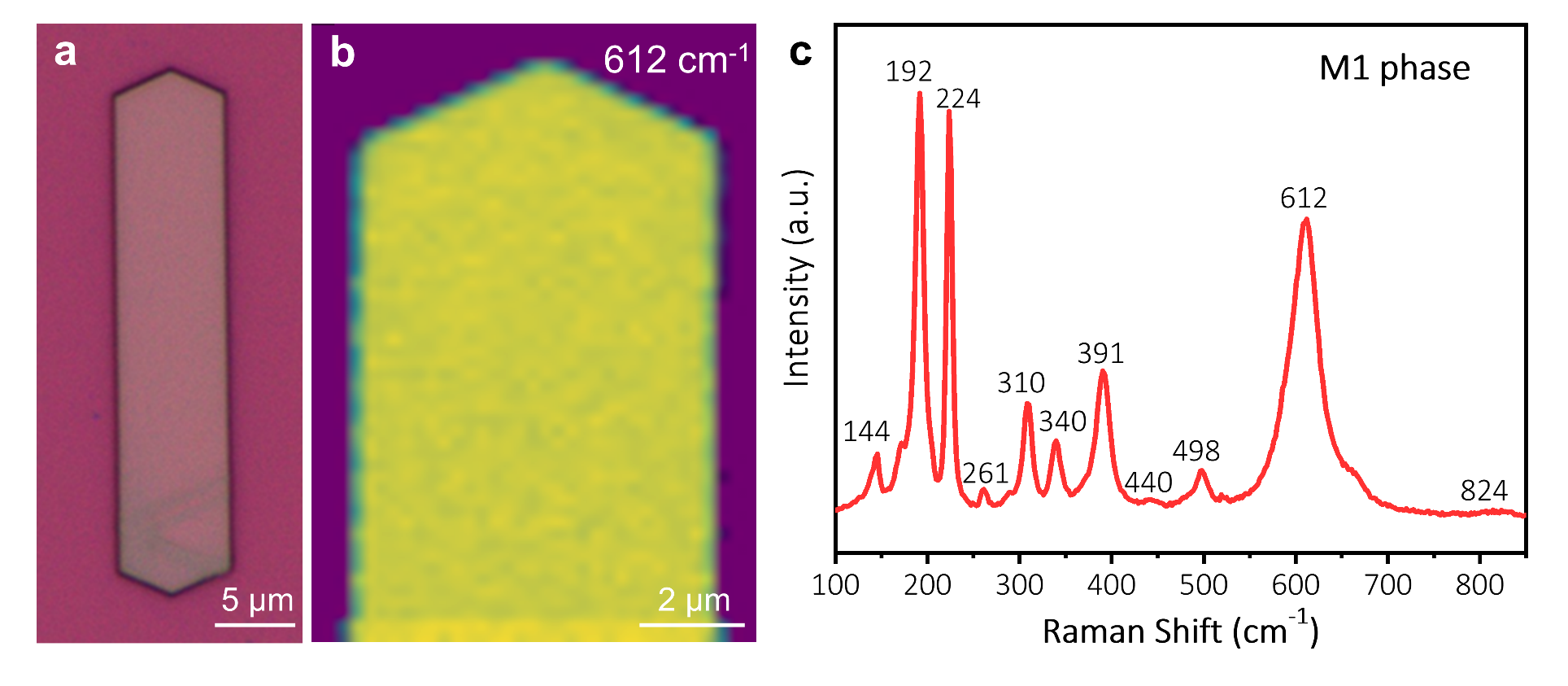
**

**Figure S4. Raman spectroscopy of thick VO_2_ nanoflake.** a) Optical image of a thick VO_2_ nanoflake and b) the corresponding Raman mapping with a peak of 612 cm^-1^. c) Raman spectrum of the thick VO_2_ nanoflake.

1. **Verifying the Raman spectrum of M_2_ phase on bent VO_2_ nanoflake**

To verify that the stripes with wrinkles in a thin VO_2_ nanoflake are in the M_2_ phase, we transferred a thick VO_2_ nanowire without any stripes and wrinkles on a flexible PEN substrate and applied different degrees of tension onto the sample (**Fig. S5a**). Optical images of the sample with and without stress were similar (**Fig. S5b**), but the corresponding Raman spectra clearly exhibited an abrupt change at a certain curvature, wherein the vibrational mode of ɷ_o_ demonstrated a remarked blue shift and the vibrational modes of ɷ_1_ and ɷ_2_ showed a slight blue shift (**Fig. S5c**). The corresponding Raman peaks of a sample under large tension were the same as the spectrum of the M_2_ phase stripes in a thin VO_2_ nanoflake, concurring with related reports[^2^](#_ENREF_2) and confirming that the Raman spectrum indeed belongs to the M_2_ phase. All the Raman spectra were collected under a Raman microscope at the same point in the VO_2_ sample (**Fig. S5d&e**).

**
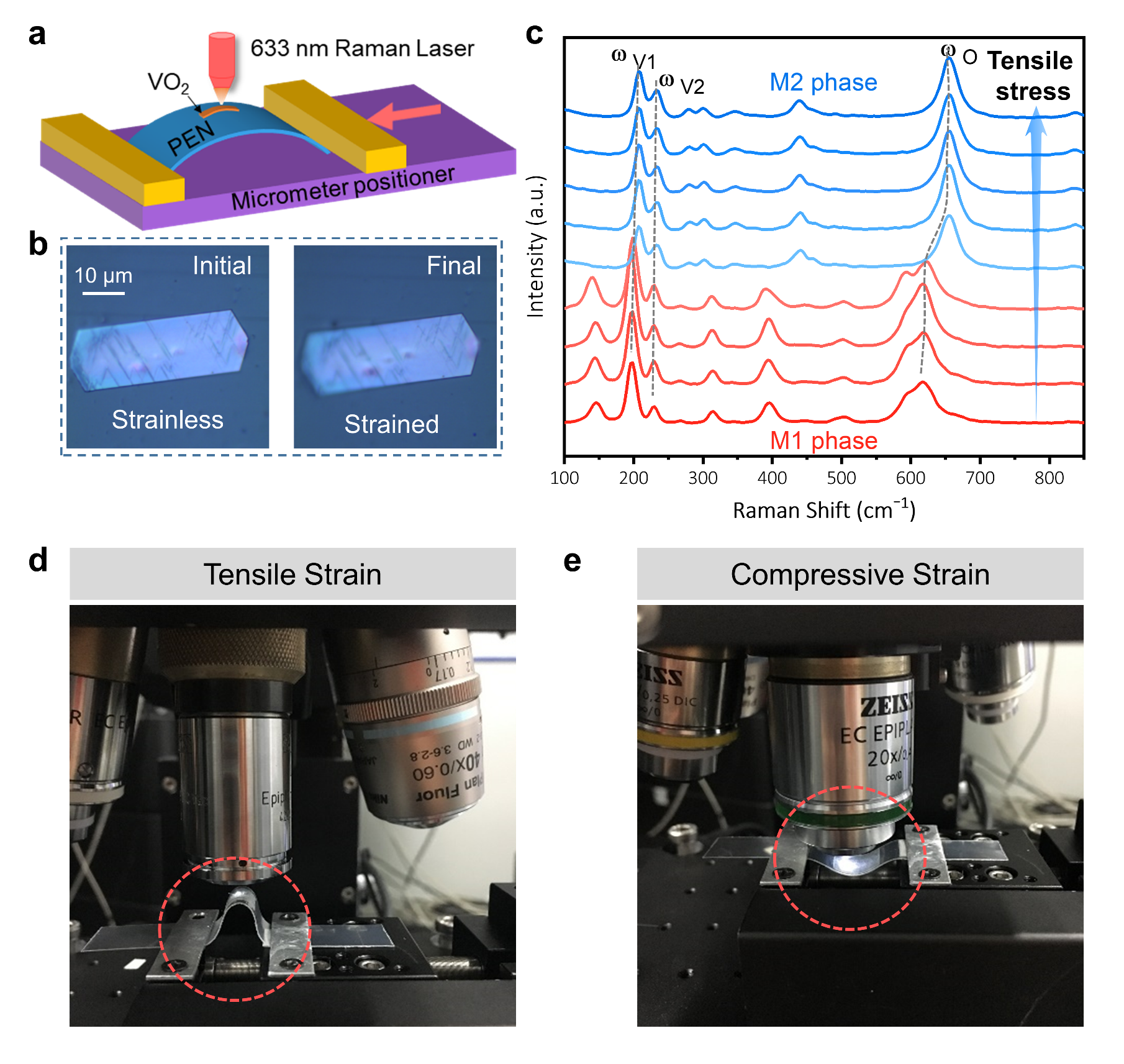
**

**Figure S5. Raman spectrum of bent VO_2_ nanoflake.** a) Schematic illustration of the configuration of Raman test. b) Optical images of a VO_2_ nanoflake before and after bending. c) Evolution of the Raman spectra under various degrees of tension. d, e) Two kinds of curvature achieved by changing the direction of bending.

1. **The phase transition path in thin VO_2_ nanoflake during heating**

The thin VO_2_ nanoflake showed piano keyboard-like alternant M_1_/M_2_ phases at room temperature (300 K). With increasing temperature, the M_1_ phase gradually transformed into the R phase, which became observable upon reaching 335 K. After the M_1_ stripes completely turned into R stripes, the M_2_ stripes began shrinking with further increase in temperature (from 350 K to 400 K).

**
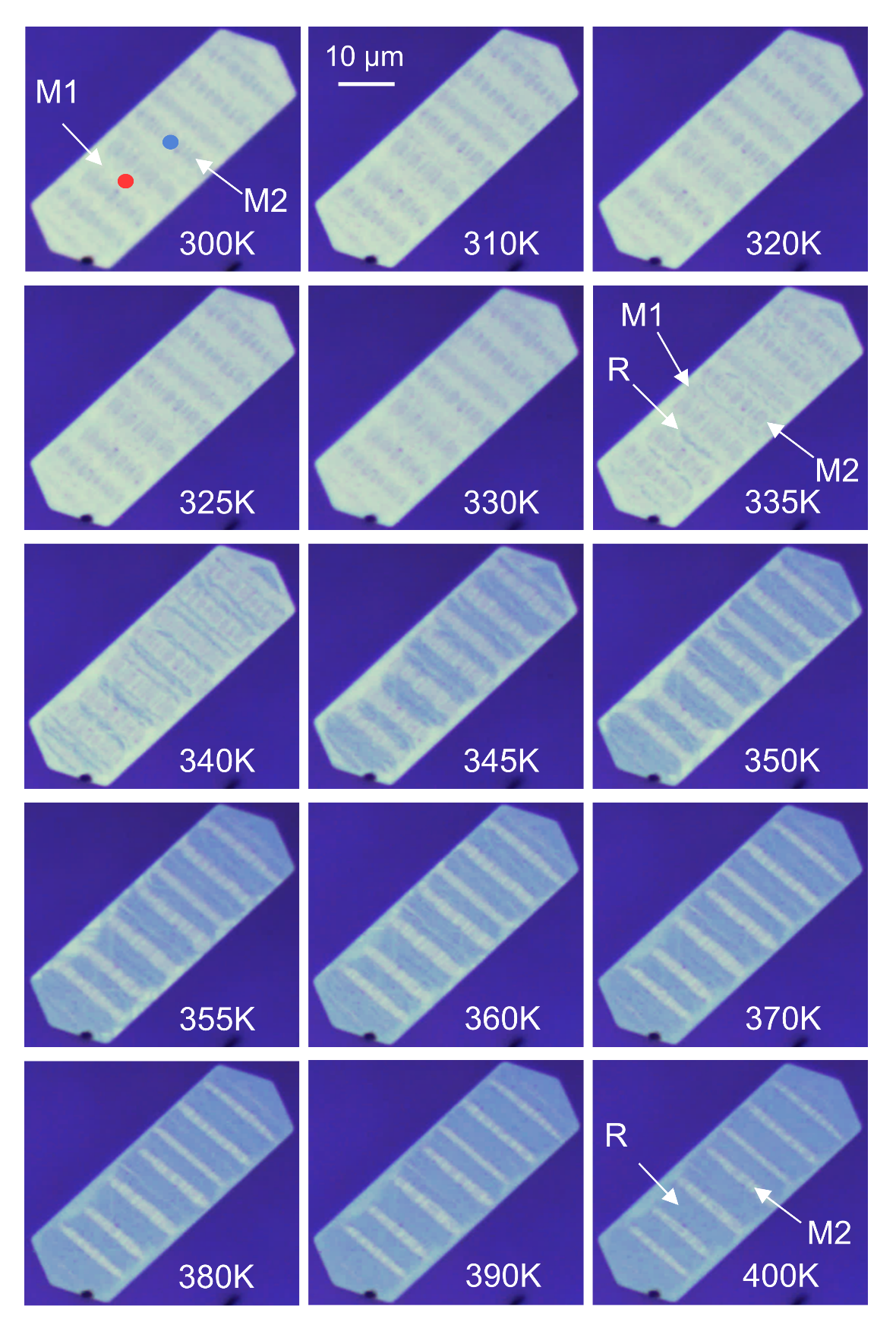
**

**Figure S6. Optical images of the evolution of the thin VO_2_ nanoflake in the temperature range from 300 K to 400 K.**

**
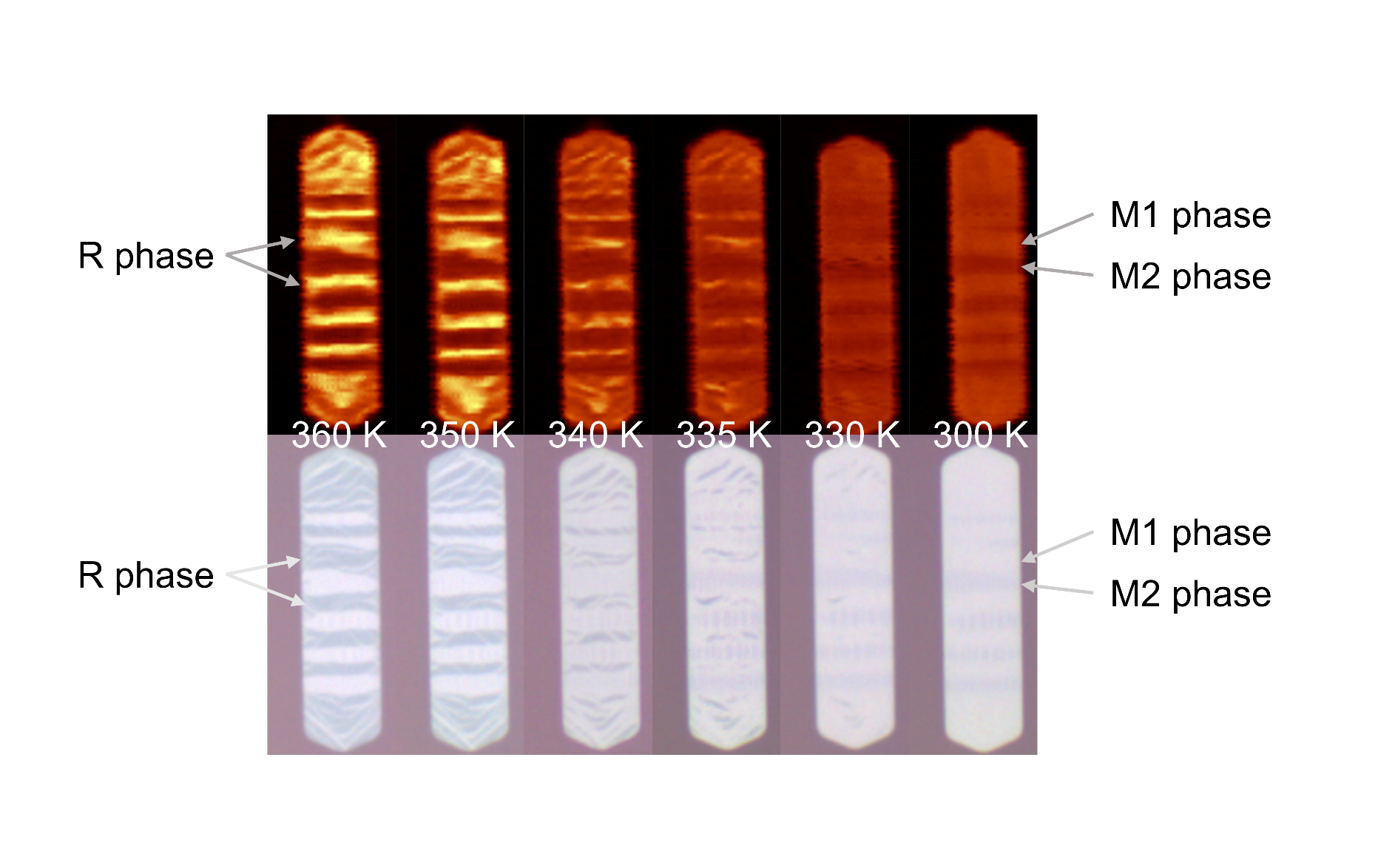
**

**Figure S7. Infrared reflection mapping (1500 nm, top) and the corresponding optical images (bottom) on another striped thin nanoflake in a temperature range between 300 K and 360 K.**

As we know, the metallic R phase and the insulating M phase showed a much larger difference at the infrared region than the visible region. The infrared reflection spectrum can therefore render a stronger contrast for distinguishing the distribution of the R and the M phases in VO_2_ nanoflakes than the common optical microscope. In **Fig. S7**, the evolution of the phase distribution in VO_2_ within a temperature range from 300 K to 360 K was compared between an optical image and an infrared reflection image and was consistent with the trend predicted by our theoretical simulation.

1. **The phase transition path in thick VO_2_ nanoflake during heating**

A thick VO_2_ nanoflake showed a different phase transition path from the thin sample. Only the M_1_ phase could be detected at room temperature. With increasing temperature, the appearance of the R phase at some locations occurred starting at 325 K. These R phase stripes grew and eventually merged to form a continuous phase covering the whole nanoflake at 360 K.

**
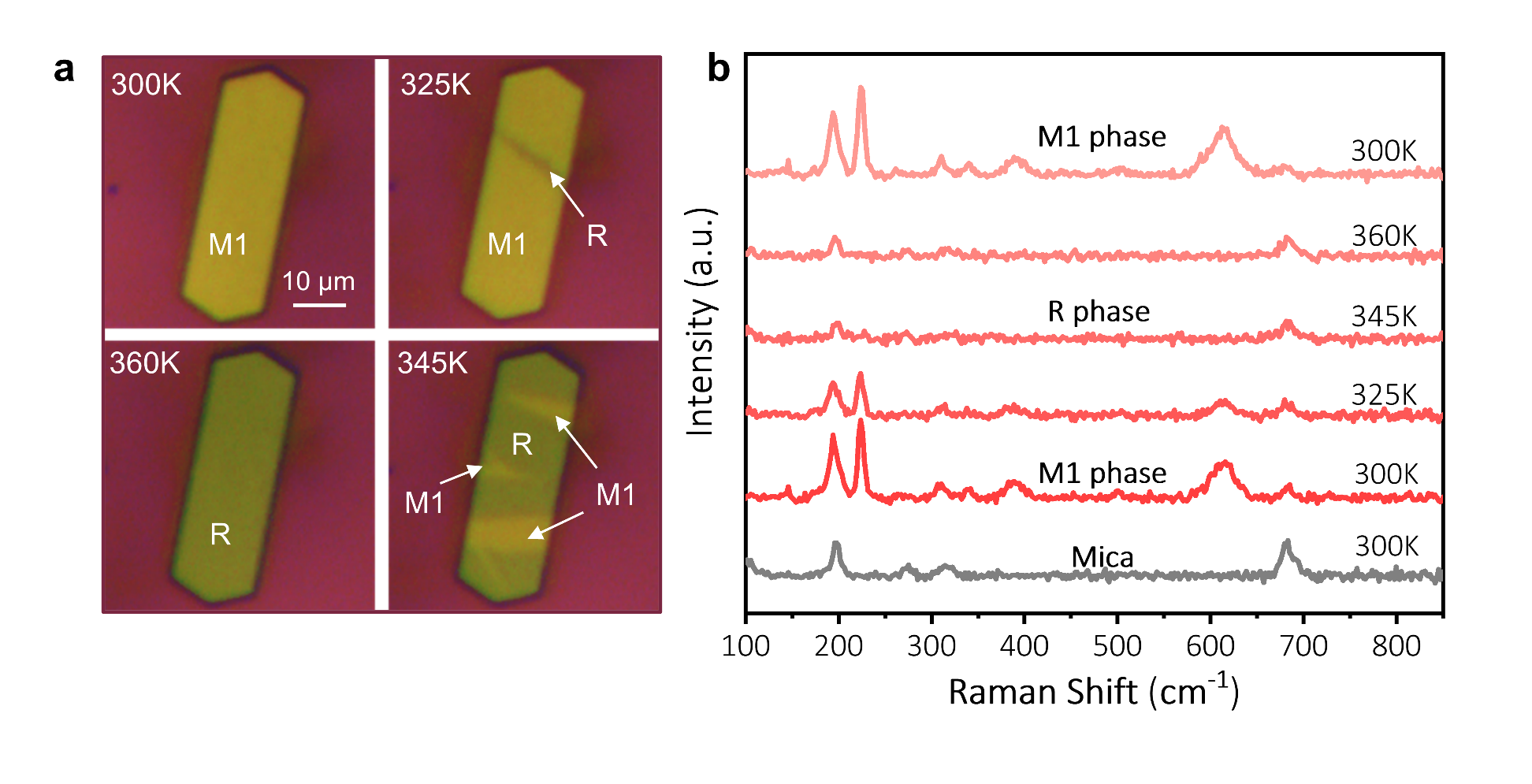
**

**Figure S8. Raman spectroscopy of the thick VO_2_ nanoflake.** a) Optical image of a thick VO_2_ nanoflake on mica under different temperatures and b) the corresponding Raman spectra of different phases (distinguished by color contrast) in the thick VO_2_ nanoflake.

1. **Comparison of the phase transition paths in thick and thin VO_2_ nanoflakes**

Phase transition paths in thick and thin VO_2_ nanoflakes exhibited two different modes. In the thick sample, the appearance and disappearance of the R phase were a random event, wherein the location of the transformed phase and the orientation of the R phase distribution were impossible to predict. In contrast, the phase transition in the thin sample was strictly restricted initially in the M_1_ stripes but eventually extended to the M_2_ stripes. The orientation of the phase distribution also demonstrated a fixed orientation perpendicular to the long axis of the VO_2_ nanoflake.

**
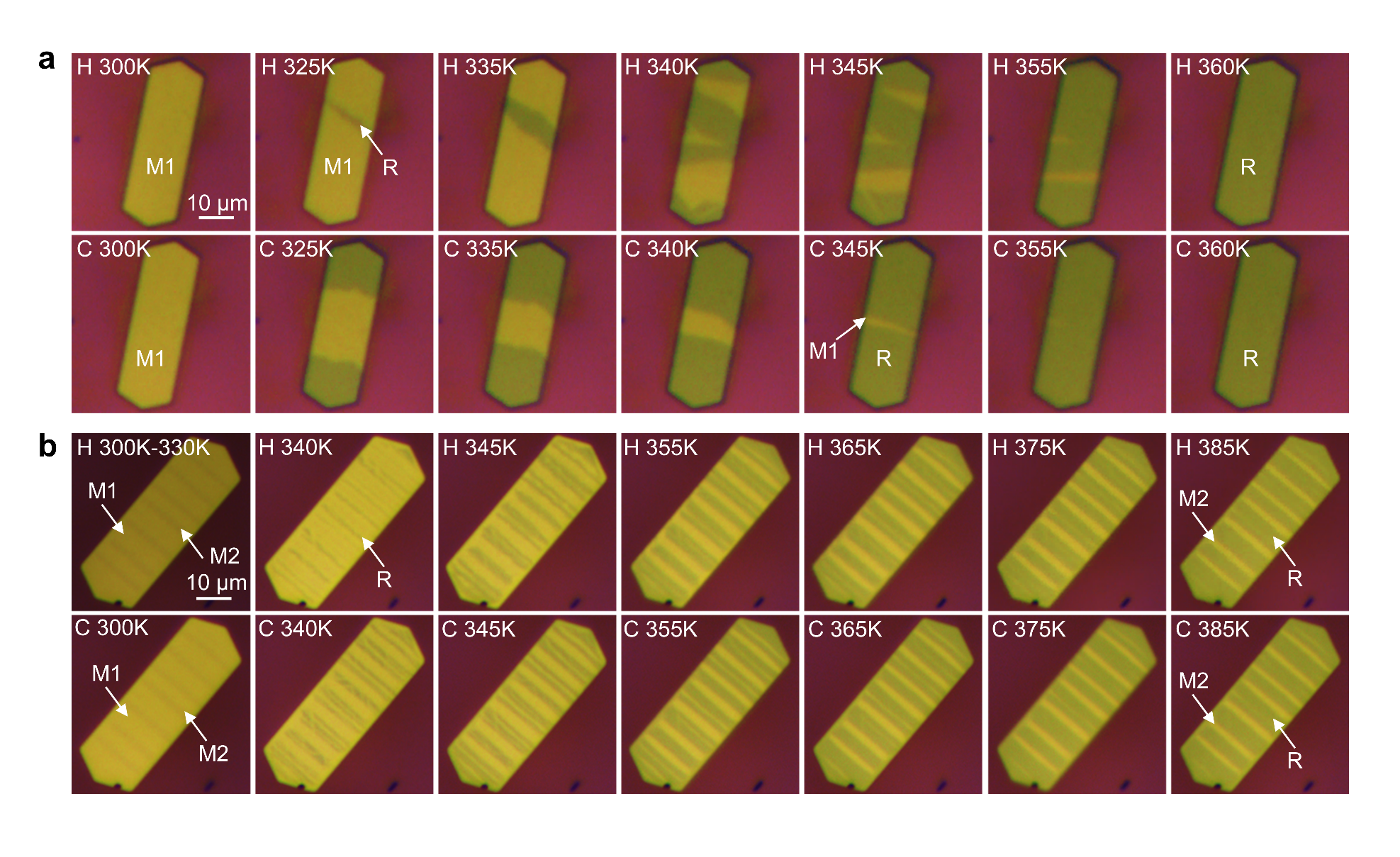
**

**Figure S9. Optical image of phase transition path induced by temperature.** a, b) Phase transition of a typical as-synthesized thick VO_2_ (pure M_1_ phase) and a thin VO_2_ (M_1_ and M_2_ phase) nanoflakes on mica during the heating and the cooling processes, respectively.

[
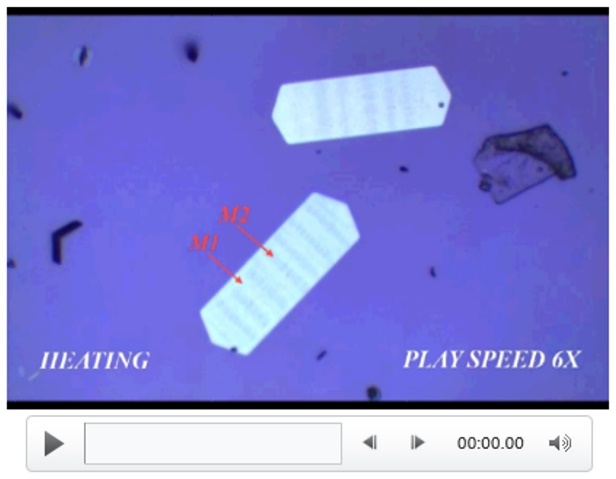
](file:///D:\00_UNISTWorkspace\15_VO2andPerovskitesonMica\Video%20S1-heating.jpg)[
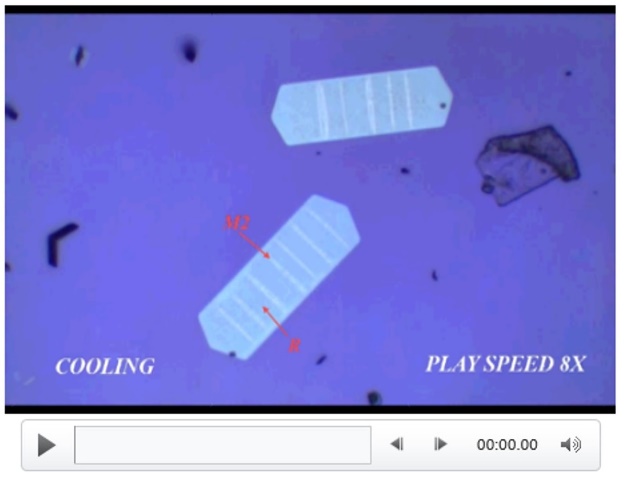
](file:///D:\00_UNISTWorkspace\15_VO2andPerovskitesonMica\Video%20S1-Cooling.jpg)

**Video S1.** In-situ optical microscopy to visualize the real-time phase transition dynamics in thin striped VO_2_ flakes during heating (300K~400K) and cooling (400K~300K), respectively.

1. **Phase transition path in transferred thin VO_2_ nanoflake on SiO_2_/Si**

For the thin VO_2_ nanoflake transferred on SiO_2_/Si, all stripes and wrinkles disappeared at room temperature and displayed typical characteristics of the M_1_ phase in the Raman spectra. During the heating process, the color of the sample gradually turned dark, indicating the phase transition from M_1_ to R. It should be noted that the appearance of the R phase was in a random manner, unlike the predictable orientation and distribution of thin samples grown on mica.

**
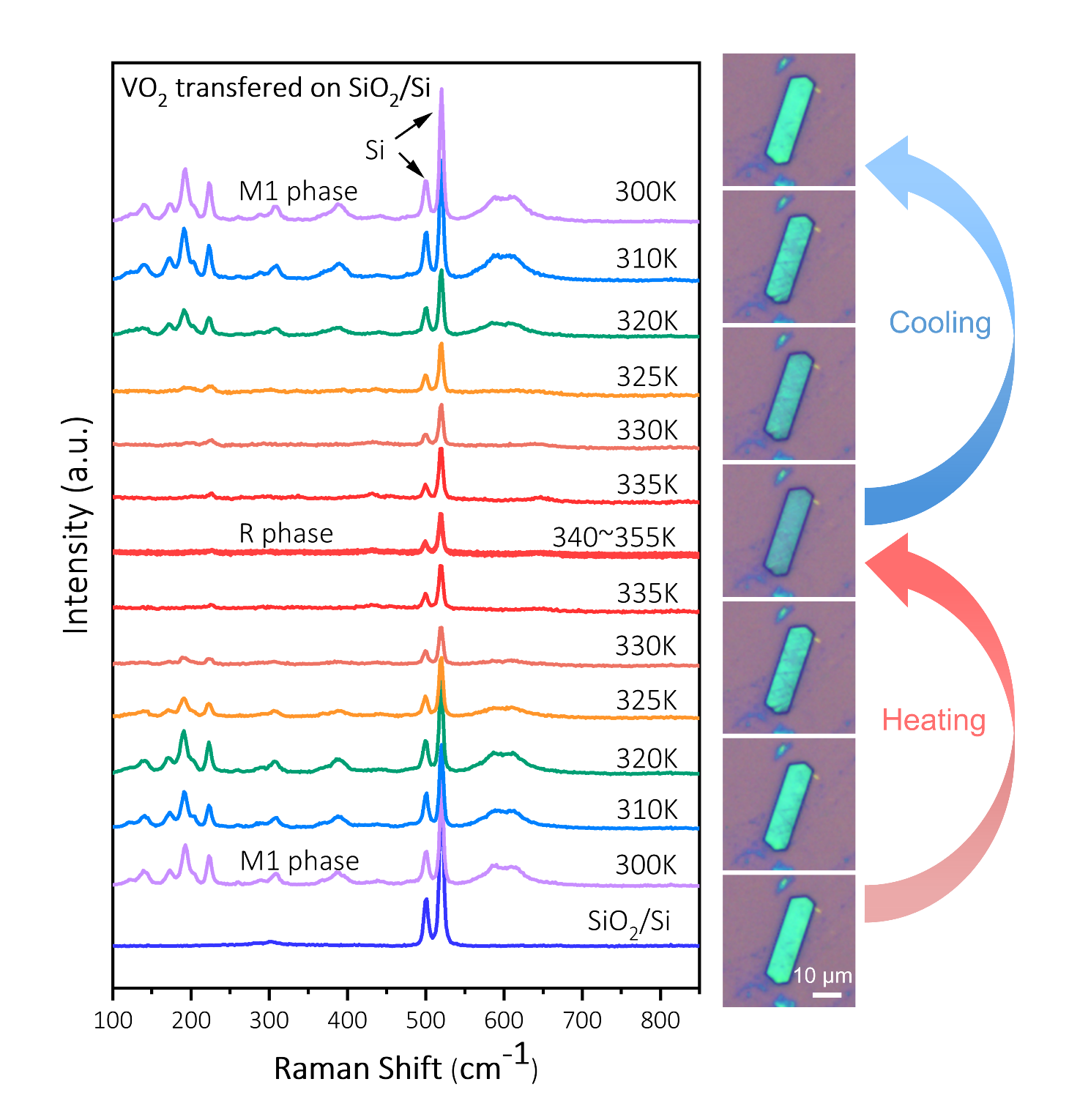
**

**Figure S10. Raman spectra of the transferred thin VO_2_ nanoflake under varied temperatures.**

1. **Phase transition induced by Raman laser in thin VO_2_ nanoflake**

Similar to the phase transition triggered by heating, Raman laser could also induce phase transition in the thin VO_2_ nanoflake. The Raman spectra under varied laser power were collected for bright (M_1_ phase) and dark (M_2_ phase) stripes. The Raman characters of the M_1_ phase disappeared at 1.5 mW, indicating the phase transition from the M_1_ phase to the R phase. In contrast, the Raman spectrum of the M_2_ phase did not change until 2.5 mW, at which point the signal of the R phase replaced that of the M_2_ phase. This result coincided with the temperature-triggered phase transition mentioned in the manuscript, which confirmed that the M_2_ phase was more stable than the M_1_ phase in the presence of interfacial stress.

**
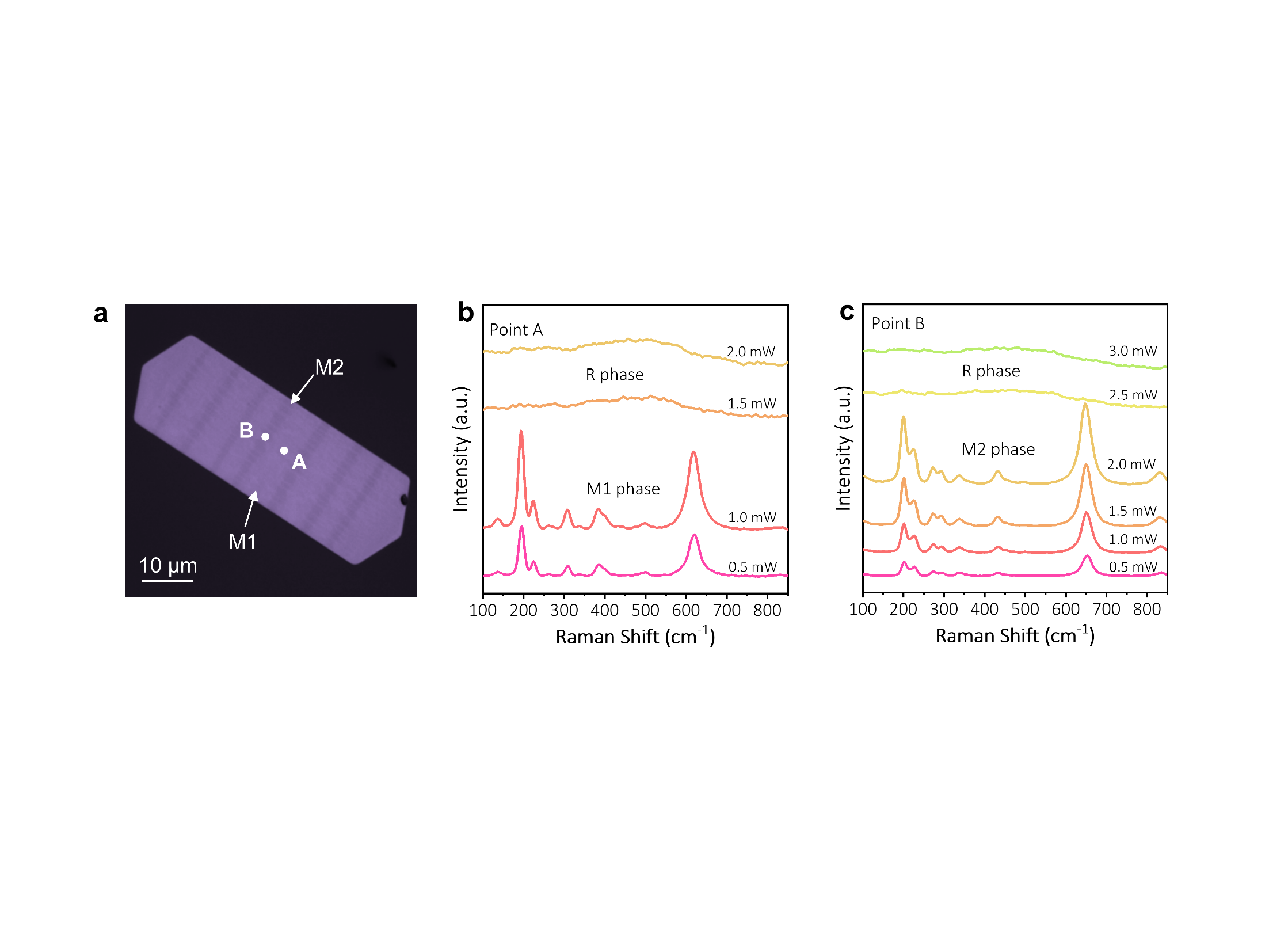
**

**Figure S11. Evolution of the Raman spectra of thick and thin samples at different laser powers.** a) Optical image of the as-synthesized thin VO_2_ nanoflake on mica substrate. b, c) Raman spectra of the M_1_ phase (bright stripe, point A) and the M_2_ phase (dark stripes, point B) at different magnitudes of laser power.

1. **Theoretical models and finite element method (FEM) simulation of stripes and wrinkles in VO_2_ nanoflakes**

**11.1 Explanation details of phase transition in thin VO_2_ nanoflakes**

The total energy for R-M_2_-M_1_ transition was expressed as follows:

$E_{T}=\alpha_{1}E_{R}\left( T \right)+\alpha_{2}E_{M_{1}}\left( T \right)+\left( 1-\alpha_{1}-\alpha_{2} \right)E_{M_{2}}\left( T \right)$, (1)

where the $E_{R}\left( T \right), E_{M_{1}}\left( T \right)$ and $E_{M_{2}}\left( T \right)$ were defined in the following form:

$E_{R}\left( T \right)=E_{R_{0}}\left( T \right)+\frac{1}{2}Y_{R}\varepsilon_{R}^{2}\left( T \right)V$ (2)

$E_{M_{1}}\left( T \right)=E_{M_{10}}\left( T \right)+\frac{1}{2}Y_{M_{1}}\varepsilon_{M_{1}}^{2}\left( T \right)V$ (3)

$E_{M_{2}}\left( T \right)=E_{M_{20}}\left( T \right)+\frac{1}{2}Y_{M_{2}}\varepsilon_{M_{2}}^{2}\left( T \right)V$ (4)

In Equation (2-4), $E_{R_{0}}\left( T \right)$, $E_{M_{10}}\left( T \right)$, and $E_{M_{20}}\left( T \right)$ denote the bulk free energy of the R, M_1_, and M_2_ phases, respectively. The $Y_{R}, Y_{M_{2}}, Y_{M_{1}}$ and $\varepsilon_{R}\left( T \right), \varepsilon_{M_{2}}\left( T \right), \varepsilon_{M_{1}}\left( T \right)$ are the Young's modulus and the strain of the R, M_2_, and M_1_ phases, respectively. The bulk free energy difference among them is described as a linear function of temperature (*T*),

${E_{M_{20}}\left( T \right)-E}_{R_{0}}\left( T \right)=\beta_{1}+\gamma_{1}T$ (5)

${E_{M_{20}}\left( T \right)-E}_{M_{10}}\left( T \right)=\beta_{2}+\gamma_{2}T$, (6)

in which $\beta_{1}$, $\beta_{2}$, $\gamma_{1}$, $\gamma_{2}$ are the parameters for the description of the bulk free energy difference near the triple point and were obtained by fitting the phase diagram (Triple point, T = 65.0 ± 0.1℃, the slopes of R-M_2_, R-M_1_, M_2_-M_1_ phase transition boundary were 29 MPa℃^-1^, 71 MPa℃^-1^, -29 MPa℃^-1^, respectively.)^8^. The fitted values of parameters $\beta_{1}$, $\gamma_{1}$, $\beta_{2}$, and $\gamma_{2}$ were -0.258, 0.000788, 0.213, and -0.000612, respectively. To solve $\frac{\partial E_{T}}{\partial\alpha_{1}}=0$ and $\frac{\partial E_{T}}{\partial\alpha_{2}}=0$, two boundary conditions were considered, the first of which was that the length of VO_2_ should be equal to that of the mica substrate as shown in **Fig. S12**. This can be expressed as an equation:

${L\left( T \right)=L}_{R}\left( T \right)+L_{M_{2}}\left( T \right)+L_{M_{1}}\left( T \right)$, (6)

$L_{R}, L_{M_{2}}, L_{M_{1}}$, and $L$ are the lengths of the R, M_2_, and M_1_ phases and the mica at temperature *T*, respectively. The above equation can be expanded to:

$Nc_{R}\left( T_{0} \right)\left( 1+K_{m}\left( T-T_{0} \right) \right)=N_{R}c_{R}\left( T_{0} \right)\left( 1+K_{R}\left( T-T_{0} \right) \right)\left( 1+\varepsilon_{R}\left( T \right) \right) +N_{M_{2}}c_{M_{2}}\left( T_{0} \right)\left( 1+K_{M_{2}}\left( T-T_{0} \right) \right)\left( 1+\varepsilon_{M_{2}}\left( T \right) \right)+N_{M_{1}}c_{M_{1}}\left( T_{0} \right)\left( 1+K_{M_{1}}\left( T- T_{0} \right) \right)\left( 1+\varepsilon_{M_{1}}\left( T \right) \right)$, (7)

where $N, N_{R}, N_{M_{2}}, N_{M_{1}}$ and $K_{m}, K_{R}, K_{M_{2}}, K_{M_{1}}$ are the number of unit cells along the growth direction and the thermal expansion coefficients of the mica and the R, M_2_ and M_1_ phases, respectively. The $c_{R}\left( T_{0} \right), c_{M_{2}}\left( T_{0} \right)$, and $c_{M_{1}}\left( T_{0} \right)$ represent the length of the unit cell along the growth direction at the growth temperature $T_{0}$ and can be defined by the following :

$c_{R}\left( T_{0} \right)=c_{R}\left( 0K \right)\left( 1+K_{R}T_{0} \right)$ (8.1)

$c_{M_{2}}\left( T_{0} \right)=c_{M_{2}}\left( 0K \right)\left( 1+K_{M_{2}}T_{0} \right)$ (8.2)

$c_{M_{1}}\left( T_{0} \right)=c_{M_{1}}\left( 0K \right)\left( 1+K_{M_{1}}T_{0} \right)$, (8.3)

in which $c_{R}\left( 0K \right), c_{M_{2}}\left( 0K \right)$ and $c_{M_{1}}\left( 0K \right)$ are the lengths of the unit cell along the growth direction at 0 K^3^. Based on references [4, 5], the phase transition relationship, the atomic structure, and the unit cell parameters of the R, M_1_, and M_2_ phases are summarized in **Table S2**. Another constraint condition was that the stress experienced by the R, M_2_, and M_1_ phases should be equal.

$Y_{R}\varepsilon_{R}\left( T \right)=Y_{M_{2}}\varepsilon_{M_{2}}\left( T \right)=Y_{M_{1}}\varepsilon_{M_{1}}\left( T \right)$ (9)

We obtained the Young's modulus and the thermal expansion coefficient of the R, M_2_, and M_1_ phases [from](#_ENREF_4) literature [6-9] (**Table S3**). Combining Equation7 to 9, the strain can be expressed as a function of the fraction of the R phase ($\alpha_{1}$), the M_1_ phase($\alpha_{2}$) and the temperature ($T$).

$$\varepsilon_{R}\left( T \right)=\{1-1.0088\alpha_{2}\left[ 1+0.000012\left( T-T_{0} \right) \right]\left( 1+0.000012T_{0} \right)/(1+$$

$$0.00003T_{0})-1.0175\left( 1-\alpha_{1}-\alpha_{2} \right)\left[ 1+0.000013\left( T-T_{0} \right) \right]\left( 1+0.000013T_{0} \right)/$$

$$\left( 1+0.00003T_{0} \right){-\alpha}_{1}\left[ 1+0.00003\left( T-T_{0} \right) \right]+0.000012\left( T-T_{0} \right)\}/\{$$

$${1.1033\alpha_{2}\left[ 1+0.000012\left( T-T_{0} \right) \right]\left( 1+0.000012T_{0} \right)}/\left( 1+0.00003T_{0} \right)+$$

$$0.91314\left( 1-\alpha_{1}-\alpha_{2} \right)\left[ 1+0.000013\left( T-T_{0} \right) \right]\left( 1+0.000013T_{0} \right)/(1+$$

$0.00003T_{0})+\alpha_{1}\left[ 1+0.00003\left( T-T_{0} \right) \right] \}$ (10)

Substituting equation (10) into the equation $\frac{\partial E_{T}}{\partial\alpha_{1}}=0$ and $\frac{\partial E_{T}}{\partial\alpha_{2}}=0$, the fractions of the R, M_2_, and M_1_ phases can be calculated and presented in **Fig. 3c**.


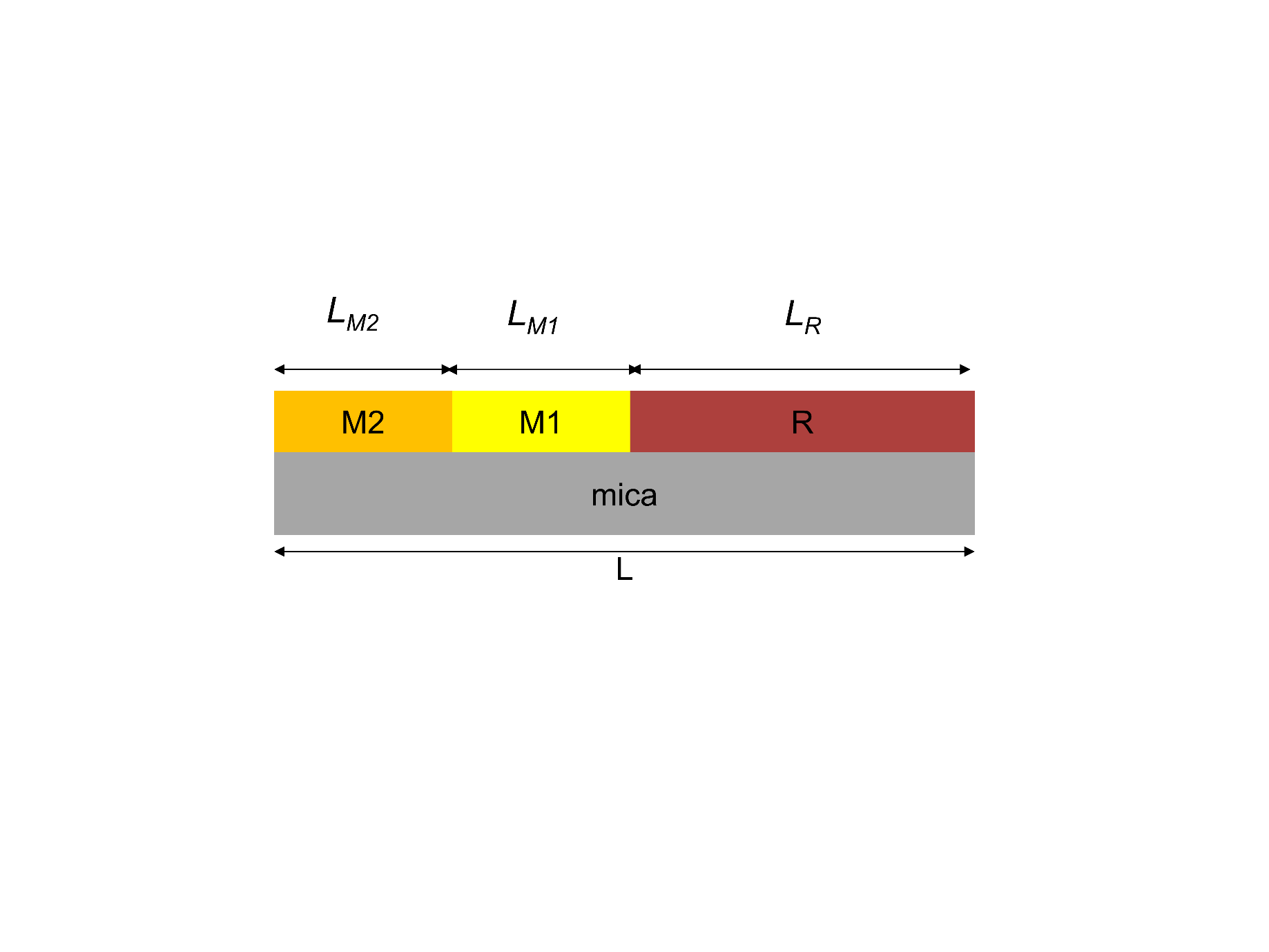


**Figure S12.** Theoretical model of the striped VO_2_ nanoflake on mica for phase fraction calculation.

**11.2 Estimation of the periods of the pattern of stripes and a relevant theoretical study about wrinkles**

**Fig. S13** shows the AFM images of VO_2_ nanoflakes grown on mica substrates with different thicknesses. From these images, we found that the periods of the pattern of stripes increased with the thickness of VO_2_ nanoflakes. The pattern disappeared when the thickness of VO_2_ nanoflakes exceeded 66 nm. **Fig. S14** presents the AFM images of the stripes and the wrinkles in a typical VO_2_ nanoflake grown on mica substrate. The average height and wavelength of the wrinkles in the M_2_ stripe were measured to be roughly 1.5 – 2 nm and about 380 nm, respectively.


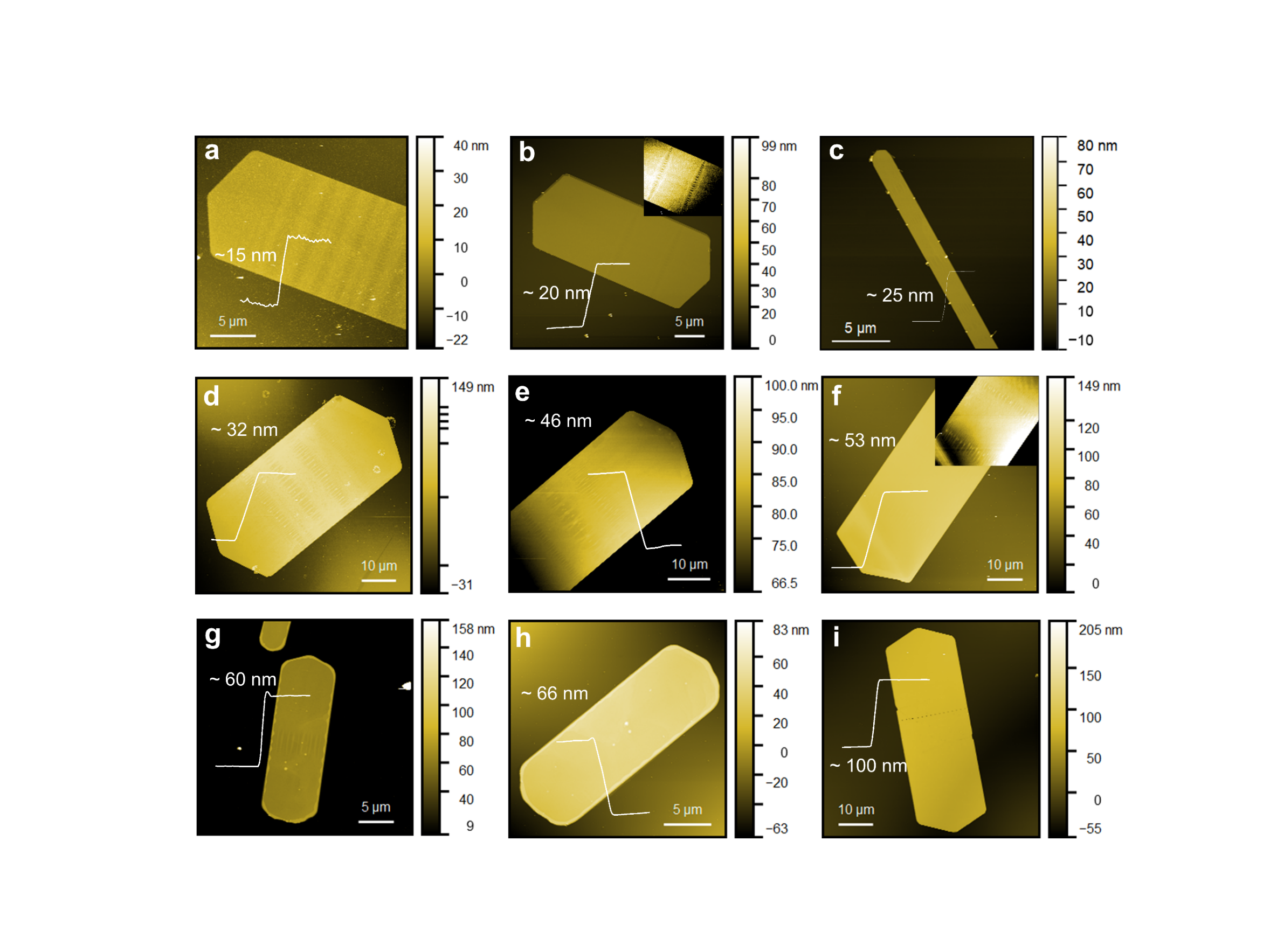


**Figure S13.** **AFM images of VO_2_ nanoflakes grown on mica substrate with different thicknesses.** a-i) The thicknesses were 15 nm, 20 nm, 25 nm, 32 nm, 46 nm, 53 nm, 60 nm, 66 nm and 100 nm, respectively.


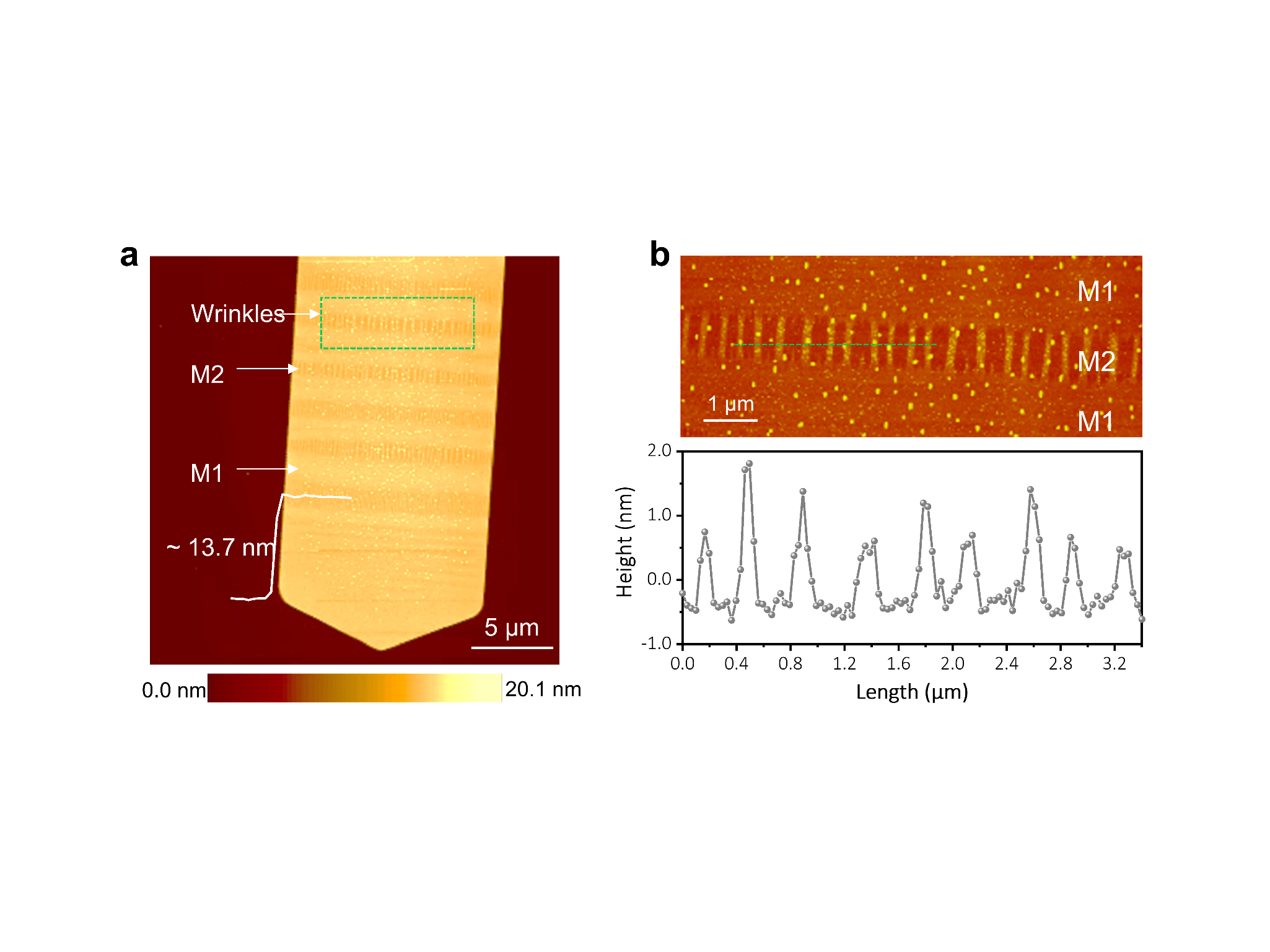


**Figure S14. AFM images of the stripes and the wrinkles in a typical VO_2_ nanoflake grown on mica.** a) AFM image of a VO_2_ nanoflake with a thickness of about 13.7 nm and M_1_/M_2_ stripes pattern. b) The corresponding AFM image of the wrinkles in the M_2_ stripe, with an average height of 1.5 to 2 nm and a period of ~ 380 nm.

- - 1. ***The estimation of the stripes patterns periods using an analytical model***

As temperature cools down, the R phase will be under tension because of the mismatch of the thermal expansion coefficients. To relax the resulting tensile stress, the R phase will slide on the mica substrate. The tensile stress was roughly assumed a linear function of the distance between the center of the R phase and the center of the M_2_ phase (The inset in **Fig. 3d**). The VO_2_ nanoflakes will eventually stop sliding when the stress caused by the frictional force equals the external tensile loading:

$\mu w\Delta L=\left[ \sigma\left( L+\Delta L \right)-\sigma\left( L \right) \right]wh$ (11)

where $w$ and $h$ are, respectively, the width and the thickness of the VO_2_ nanoflake; $\Delta L$ is a small element in the R phase; $\sigma\left( L \right)$ and $\sigma\left( L+\Delta L \right)$ are the stress magnitudes at distances $L$ and $L+\Delta L$; and $\mu$ is the frictional force per area. The simplified formula of equation (11) then becomes:

$\frac{d\sigma\left( L \right)}{dL}=\frac{\mu}{h}$ (12)

The stress at distance *L* is calculated by the integral of equation (12).

$\sigma\left( L \right)=\frac{\mu}{h}L+\sigma\left( 0 \right)$ (13)

Based on the following two boundary conditions,

$L=0: \sigma\left( 0 \right)=Y_{R}\varepsilon_{0}$ (14.1)

$L=\pm\frac{L_{c}}{2}: \sigma\left( \pm\frac{L_{c}}{2} \right)=0$ (14.2)

where $\varepsilon_{0}$ is the tensile strain when the temperature cools down to ~ 440 K, the relationship between the periods of the pattern (*L_c_*), and the frictional shear stress (*u*), the thickness (*h*) was established in the following form:

$L_{c}=\frac{2Y_{R}\varepsilon_{0}h}{u}$ (15)

***11.2.2 Critical thickness estimation for wrinkle formation***

For **Fig. S15**, the wavelength (λ) and the amplitude (*h*) of the wrinkle was previously determined. Based on the profile of the wrinkles, an analytical model is proposed in **Fig. S15**. The curvature of the wrinkle (*r*) was calculated to be ~ 6017 nm using the following equation:

$\left( r-h \right)^{2}+{(\lambda/4)}^{2}=r^{2}$ (16)

The dimensions of the unit cell are summarized in **Table Sa**. At 440 K, the length of the unit cell along the *x*-direction (appendicular to growth direction) were calculated using the following equations,

$x_{R}\left( T \right)=x_{R}\left( 0K \right)\left( 1+K_{R}T \right)$ and $x_{M_{2}}\left( T \right)=x_{M_{2}}\left( 0K \right)\left( 1+K_{M_{2}}T \right)$ (17)

The length of the R phase along the *x*-direction should be about 0.34% shorter than that of the M_2_ phase to induce wrinkles in the M_2_ phase. Thus, the compressive strain energy ($E_{c}$) should be greater than the bending energy ($E_{b}$) to form a wrinkle. The compressive strain energy and bending energy are described in the following forms:

$E_{c}=\frac{1}{2}Y_{R}\varepsilon^{2}whl$ (18)

$E_{b}=\frac{1}{2}\frac{Y_{R}h^{3}}{12\left( 1-v^{2} \right)}\left( \frac{1}{r} \right)^{2}wl$, (19)

where $Y_{R}$, $\varepsilon$, $v$, $w$, and $l$ denote the Young’s modulus, the compressive strain, the Poisson’s ratio, and the width and the length of the R phase, respectively.

**
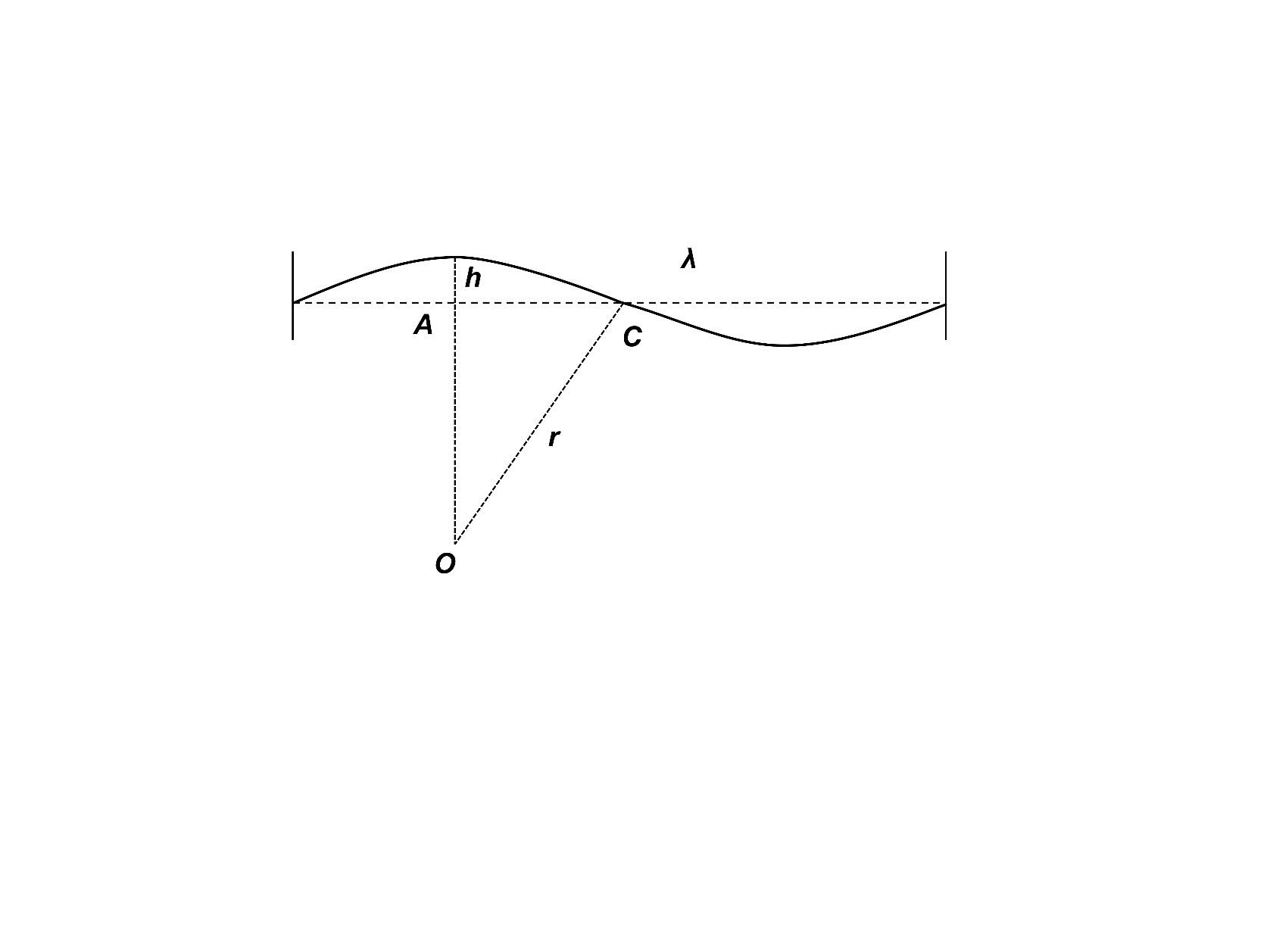
**

**Figure S15.** Analytical model for estimating the critical thickness for wrinkle formation.

***11.2.3 Finite element method simulation for wrinkle analysis***

For a better understanding of the wrinkling phenomenon, a 3D finite element model was developed using the commercial software ABAQUS^10^. The detailed descriptions of the finite element model are shown in **Fig. S16**. Based on the experimental sample (**Fig. S14**), we established the finite element model and the boundary conditions in **Fig. S16a-b**. The left surface was fixed in the y and z-directions, while the top and bottom surfaces were clamped in the x and z-directions. In **Section 11.2.2**, we elicited that the length of the R phase along *x*-direction was ~ 0.34% shorter than that of the M_2_ phase, which was applied to the model in **Fig. S16b**. The material properties used for the simulation are also presented in **Table Sb**. A linear perturbation procedure was firstly performed with *BUCKLE, in which eigenmodes with a different number of wrinkles were introduced as a geometric imperfection to initiate the VO_2_ nanoflake buckling[^11^](#_ENREF_11). The Riks method as implemented in ABAQUS was then employed for the post-buckling analysis[^12^](#_ENREF_12). As shown in **Fig. S16c**, when the wrinkle number was equal to 20, the simulated wrinkle height approximates the experimental measurement. We roughly estimated the wrinkle wavelength to be about 425 nm.


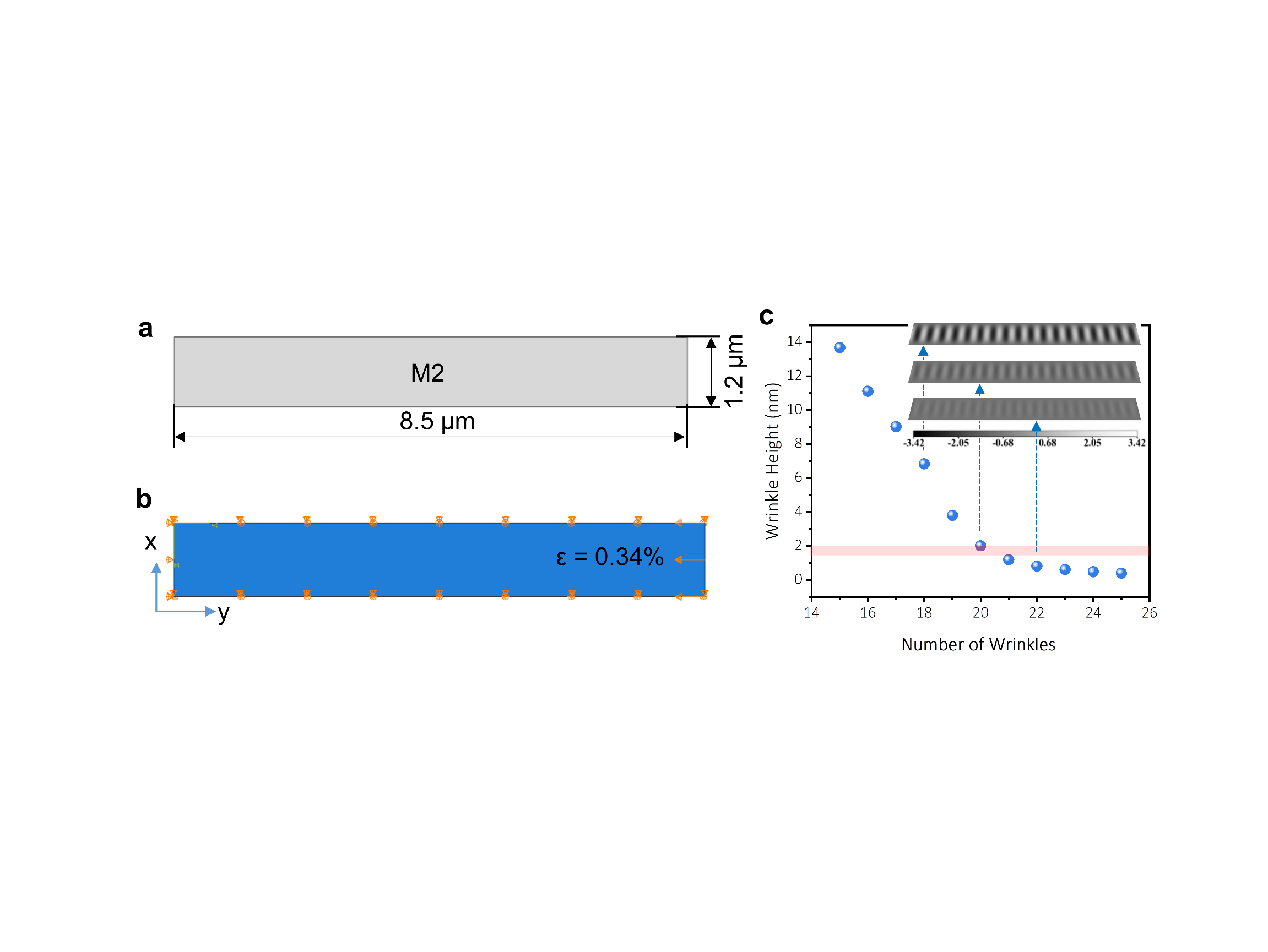


**Figure S16.** a) FEM modeling for the M_2_ phase. b) Boundary condition and loading in the FEM model. c) The wrinkle height and surface morphology variation with different wrinkle number, where the red line represents the experimentally observed wrinkle height.

**Table Sa**. The phase transition relationship, the atomic structure, and the unit cell parameters of the R, M_1_, and M_2_ phases.


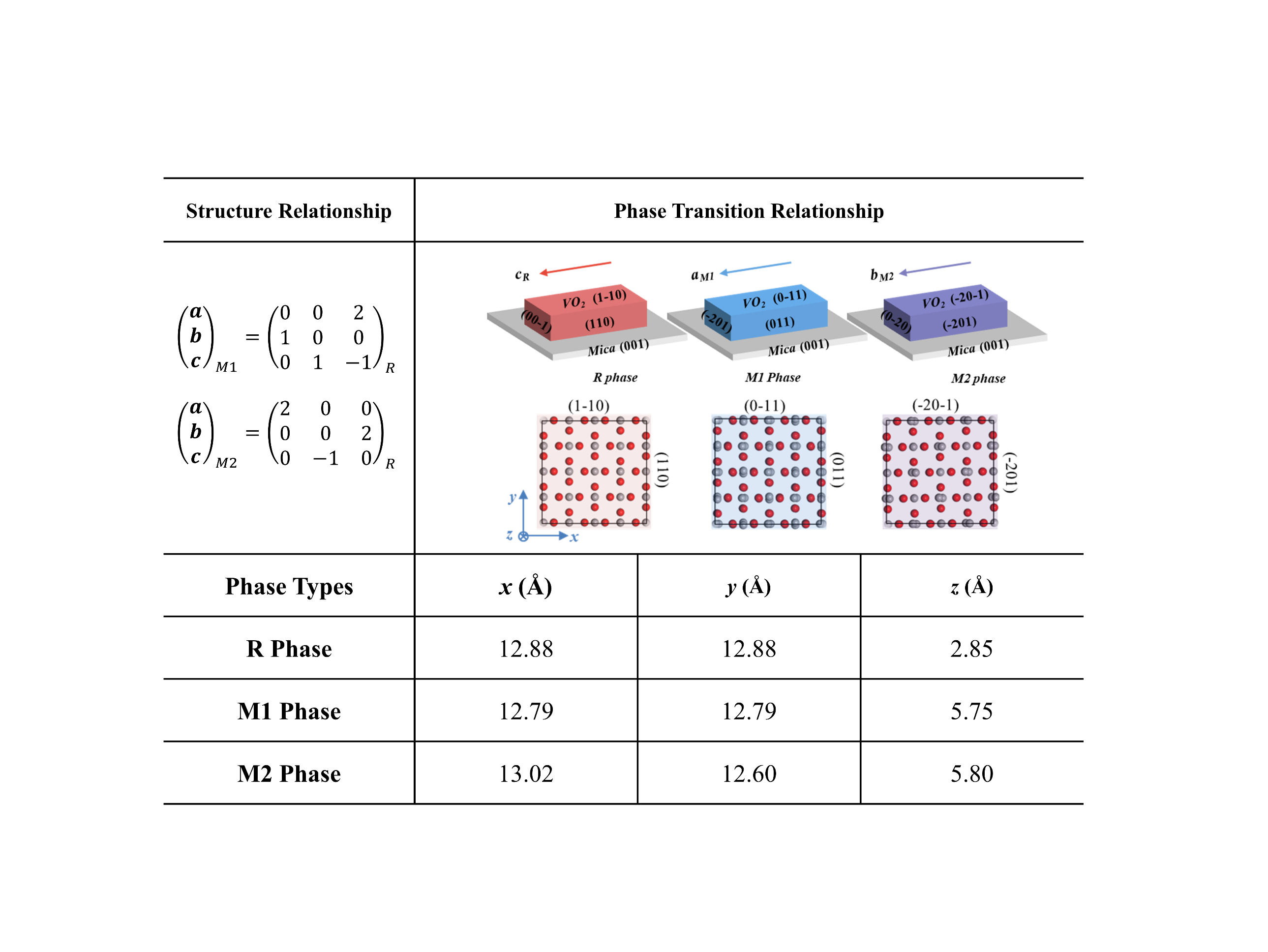


**Table Sb.** Physical parameters of R-VO_2_, M_1_-VO_2_, M_2_-VO_2_, and mica substrate ^[6-9]^.

| **Phase** | **Young’s modulus**  **(GPa)** | **Thermal expansion coefficient**  **(/K)** | **Poisson’s ratio** |
| --- | --- | --- | --- |
| R-VO_2_ | 140 | 30×10^-6^ | 0.2 |
| M1-VO_2_ | 128 | 12×10^-6^ | 0.2 |
| M2-VO_2_ | 156 | 13×10^-6^ | 0.2 |
| Mica | / | 12×10^-6^ | / |

1. **In-plane electrical anisotropy modulated by oriented phase transition**

The value of the electrical anisotropic ratio was varied case by case because of different channel parameters, such as the width of each phase, the number of phase interface, and the intensity of the interfacial stress. However, the significant enhancement of the anisotropic ratio was similar in different samples. Moreover, the final anisotropic ratio was mainly dependent on the conductance ratio of the transformed state because the initial conductance ratio of the sample was almost a constant that was independent of the variation of the sample thickness (**Table S4**).

**
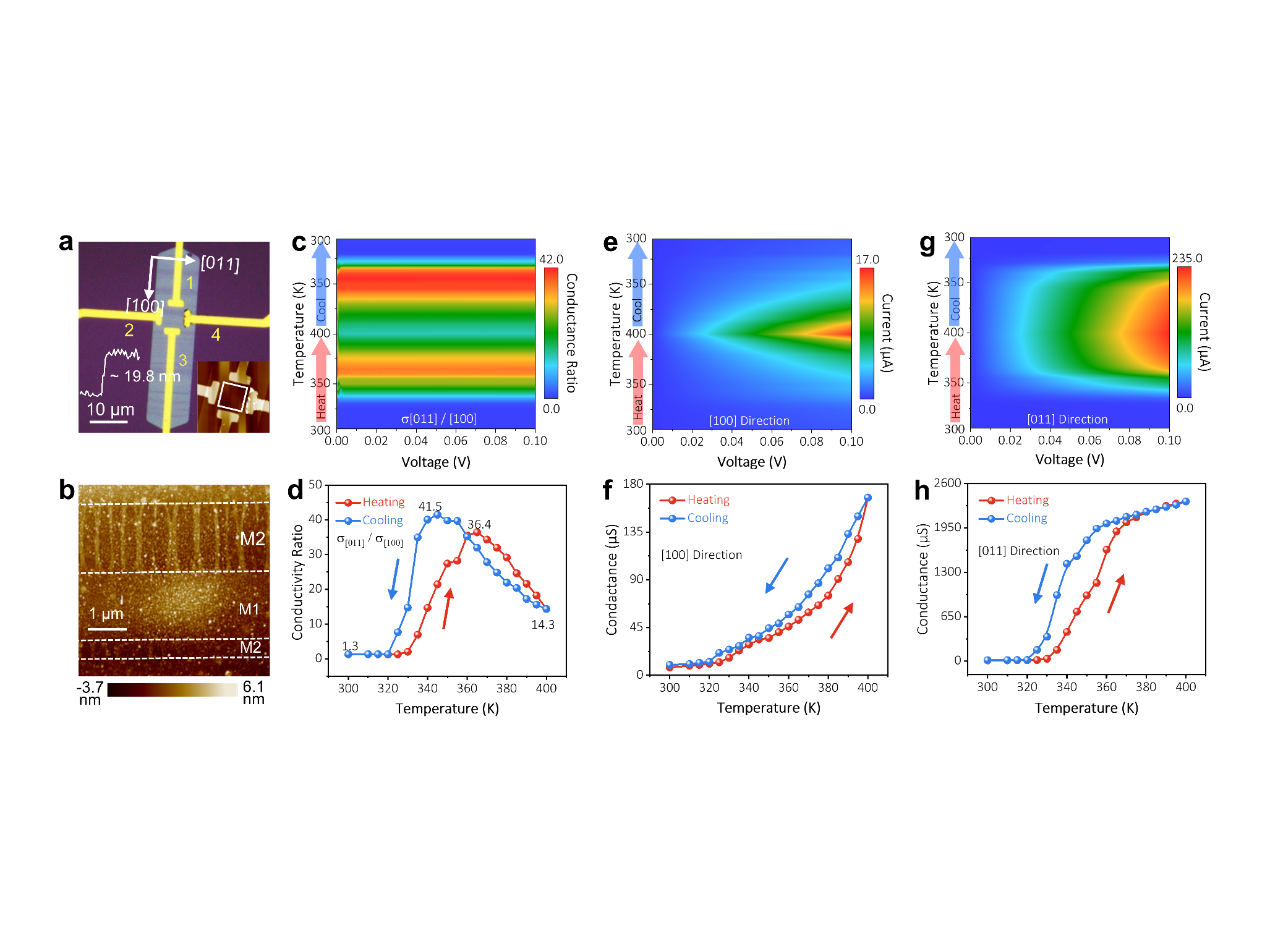
**

**Figure S17. Modulation of in-plane electrical anisotropy of thin VO_2_ nanoflake device.** a) Optical image of the thin VO_2_ nanoflake device and the corresponding AFM image showing a thickness of 19.8 nm. b) Enlarged AFM image of the solid boxed area in (a), confirming the existence of the M_1_ and M_2_ phases in the channel. c) The temperature-dependent anisotropic conductance ratio at varied voltages displayed in 2D mode and d) the anisotropic conductance ratio curves extracted from (c) at a bias voltage of 0.1 V. e, g) The temperature-dependent current of the [100] axis and the [011] axis at varied voltages, respectively. f, h) The conductance-temperature curves of tje [100] axis and the [011] axis extracted from (e) and (g), respectively. All the anisotropic electric properties were measured in ambient environment.

**Table S2.** Comparison of the initial electrical anisotropic ratio of various VO_2_ thicknesses

**
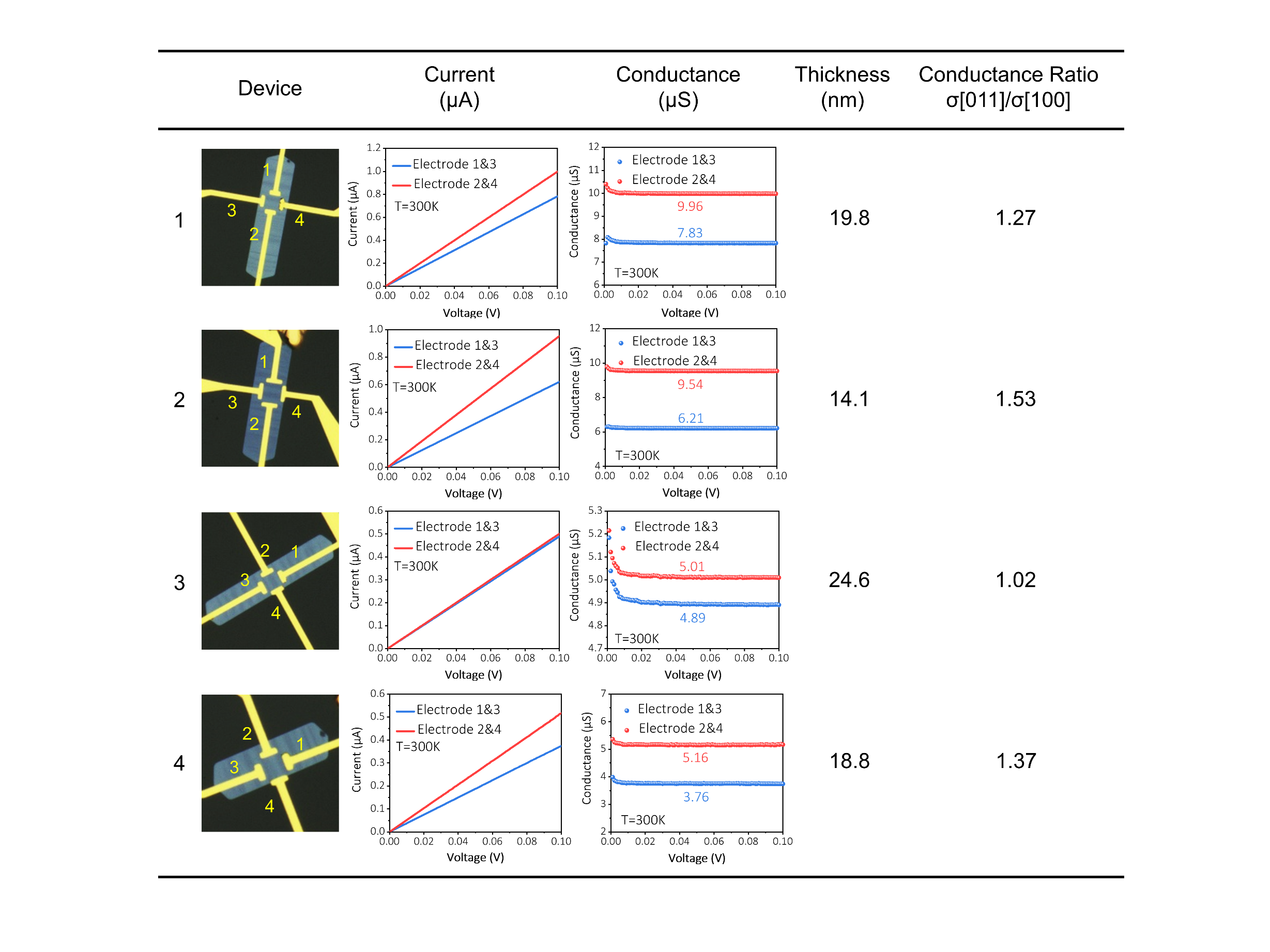
**

**
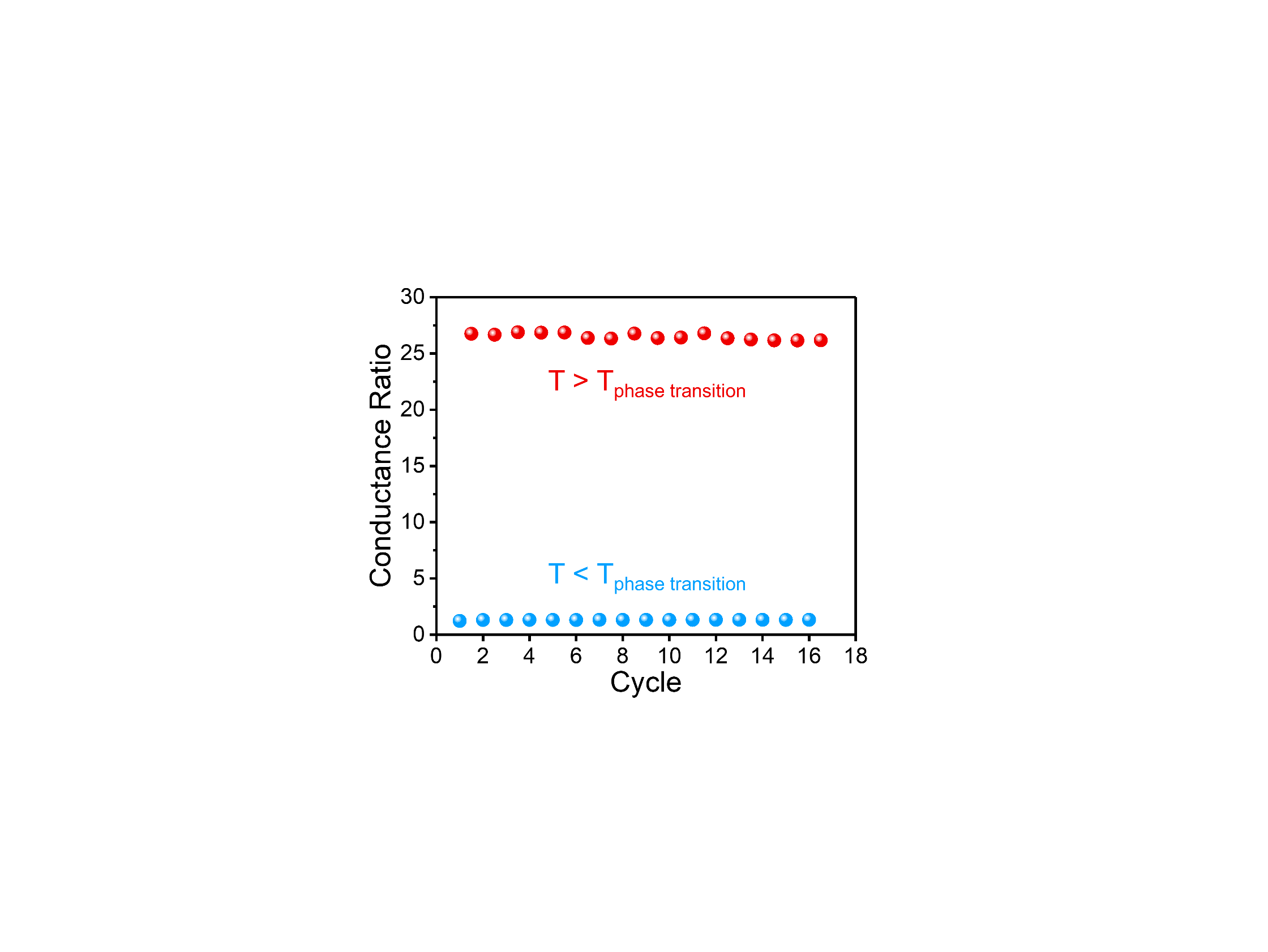
**

**Figure S18. Stability of IPA modulation under cycles of heating and cooling.**

**Table S3.** Comparison of the conductance anisotropic ratios with other common anisotropic 2D materials and with other enhanced strategies.

| Materials | In-plane conductance ratios | Remarks | Ref. |
| --- | --- | --- | --- |
| VO_2_ | 1.5-113 | Periodic phase engineering modulated | This work |
| b-As | 1.47 | Intrinsic | [13] |
| b-P | 1.5 | Intrinsic | [14] |
| b-P | ~6 | Enhanced by anisotropic atoms intercalation | [15] |
| GeAs_2_ | 1.8 | Intrinsic | [16] |
| GeP | 1.52 | Intrinsic | [17] |
| NbOI_2_ | 1.34 | Intrinsic | [18] |
| PbSnS_2_ | 1.77 | Intrinsic | [19] |
| PdSe_2_ | 2.25 | Intrinsic | [20] |
| PdPSe | 3.85 | Intrinsic | [21] |
| ReS_2_ | 2-8.2 | Gate bias modulated | [22] |
| ReS_2_ | 1.4-12 | Local ferroelectric field modulated | [23] |
| ReS_2_ | 1.17-3.41 | Doping-mediated lattice engineering modulated | [24] |
| WO_x_ | ~5 | Defects engineering enhanced | [25] |
| Te | 1-2 | Strain engineering modulated | [26] |
| Graphene | ~18 | Enhanced by anisotropic wrinkles | [27] |

1. **Model for understanding the evolution of electrical anisotropic ratio**

The conductance curves along the [011] (electrode pair 24) and the [100] (electrode pair 13) directions exhibit different behaviors. The former showed a sharp change near the phase transition temperature, while the latter demonstrated only a gradual change with temperature. The difference of the conductance between the two directions was therefore dynamically changing with temperature. This nonsynchronous change can be further understood through the following model. Taking the heating process as an example, according to the conductance curve shown in **Fig. S19a-b**, we can divide the curve to be four stages (**Fig. S19c**). In stage I (300-325 K), the M_1_ and M_2_ stripes showed no obvious change. The M_1_ and M_2_ stripes were in series along [100] but paralleled along [011], but the former demonstrated a slightly larger resistance than the latter, leading to an initial electrical anisotropic ratio ([011]/[100]) of about 1 to 2. With increasing temperature, the channel evolved into stage II (325-355 K), in which the R phase emerged in the M_1_ stripes, but the M_2_ stripes remained. In this case, the conductance increased in both directions, but [011] showed a minor advantage over the [100] direction, resulting in a gradual increase of the conductance ratio. In stage III (355-375 K), the R phase covered most of the M_1_ stripes, increasing the conductance of [011] sharplyIn contrast, the existence of M_2_ suppressed the abrupt change of the conductance along [100], resulting in the significant enhancement of the conductance ratio. With the further elevation of temperature, the R phase covered the whole area of the M_1_ stripes in stage IV, while the M_2_ stripes gradually turned into the R phase as well. In this case, the conductance in both directions increased slowly, but the increase was faster in the [100] direction, resulting in a slow decrease of conductance ratio in stage IV. Corresponding optical images of these four stages were provided in **Fig. S20a-b**. When the temperature increased further from 400 to 440 K, the M_2_ stripes kept shrinking and finally transformed into the R phase completely (**Fig. S20c**).

For the cooling process, the evolution trend was similar, but a thermal hysteresis was clearly observed because of the lattice incompatibility between the transformed phase and the parent phase. This conductance change due to phase transition was reversible as shown in **Fig. S21**. The recovery of the work current to the same level after the heating and cooling cycle can be attributed to the excellent reversibility of the phase transition in VO_2_.

**
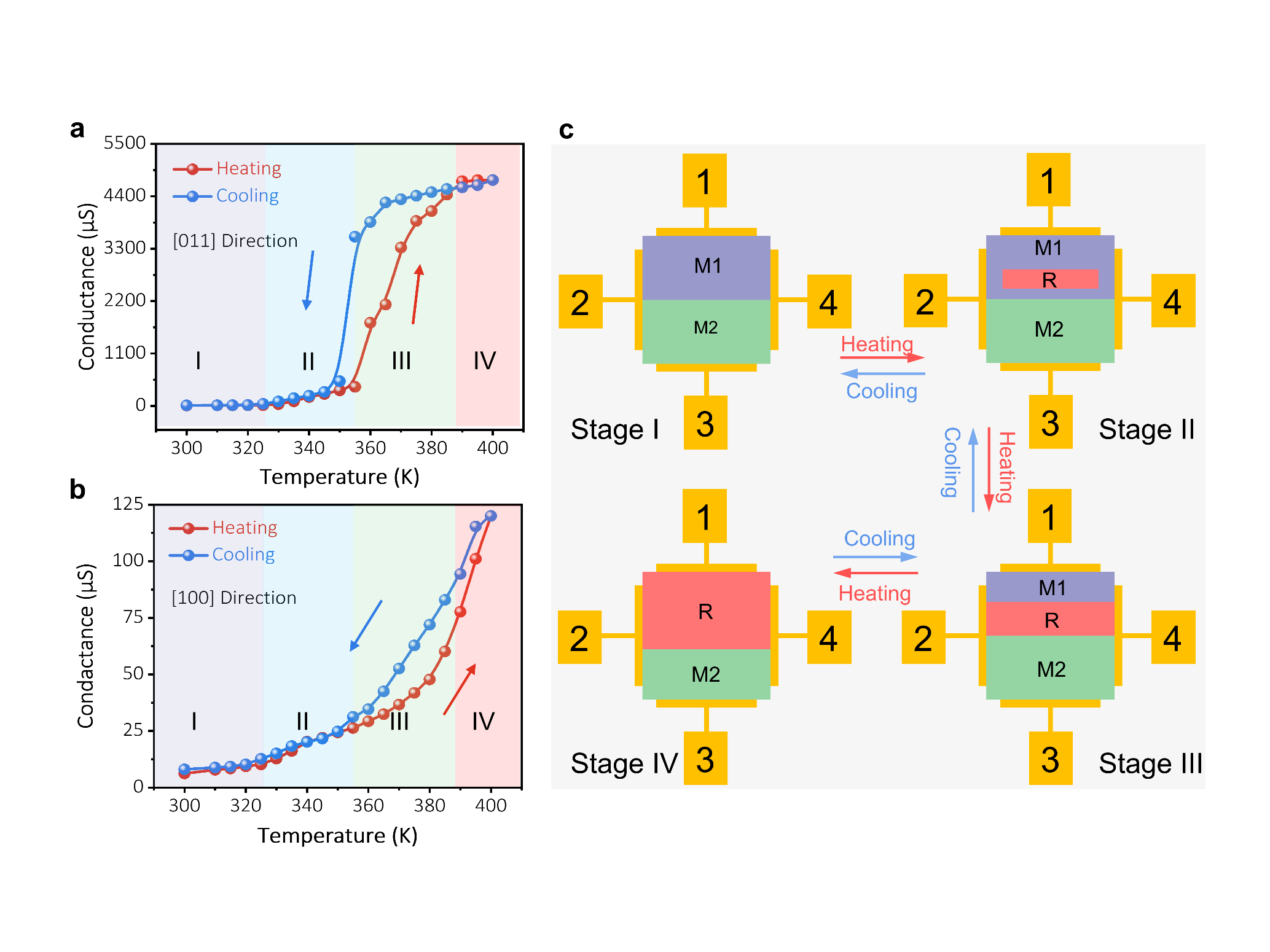
**

**Figure S19. Mechanism of the evolution of electrical anisotropic ratio in thin VO_2_ device.** a, b) Conductance-temperature curves of [011] axis and [100] axis during heating and cooling processes, respectively. c) Schematic illustrating the paths of oriented phase transition.

**
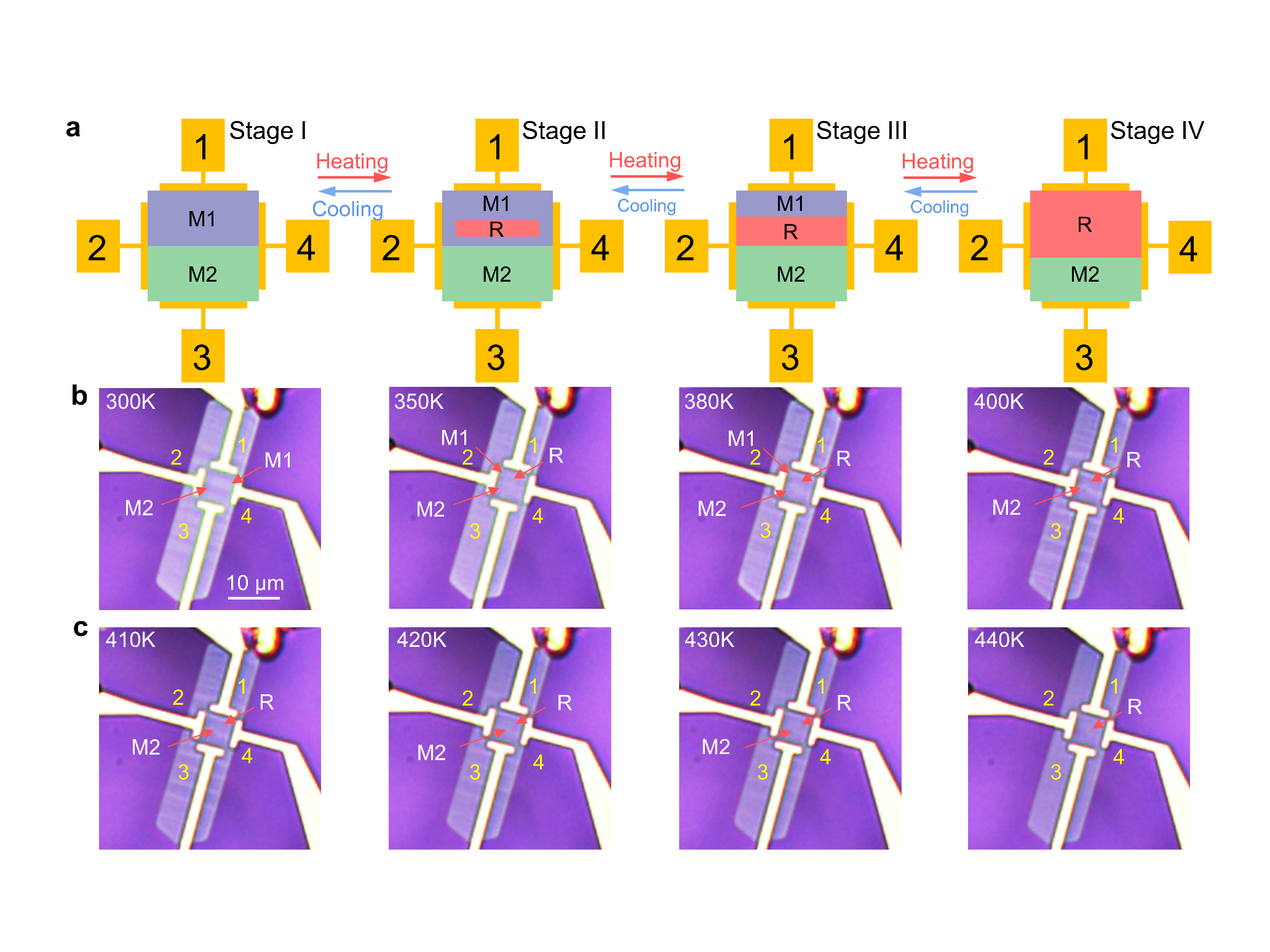
**

**Figure S20. Optical image of thin VO_2_ device during the heating process.** a) Schematic illustrating the paths of oriented phase transition. b) Optical image (300K to 400K) of the device for each stage in Figure S9. c) Optical image of the device at higher temperature (410K to 440K), which confirmed that the M_2_ phase completely transformed to the R phase.

**
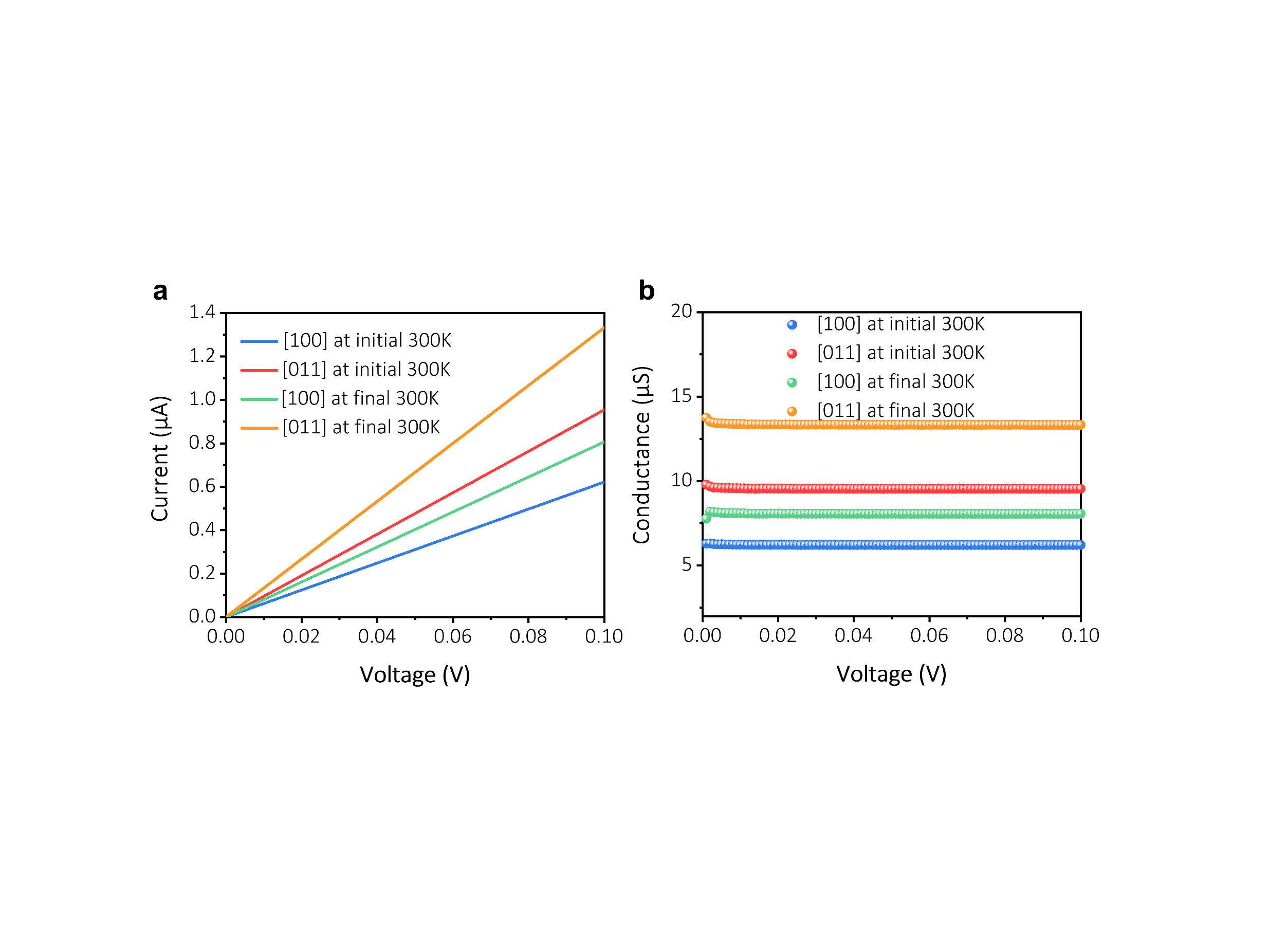
**

**Figure S21. IV curves of both directions at initial and final states.** All the current stayed at the same level.

To further understand the phase transition details in this process, we made a differential operation on the conductance curves shown in **Fig. S19a-b** to temperature. As can be seen in **Fig. S22**, a maximum value exists along the [011] direction in both heating and cooling curves, corresponding to the phase transition temperature of about 360 and about 350 K, respectively. In contrast, the derivative curve along the [100] direction showed an almost monotonously increasing trend without a maximum at the supposed phase transition temperature. In other words, a specific phase transition temperature was absent along the [100] direction as the phase transition occurred with a gradual change.


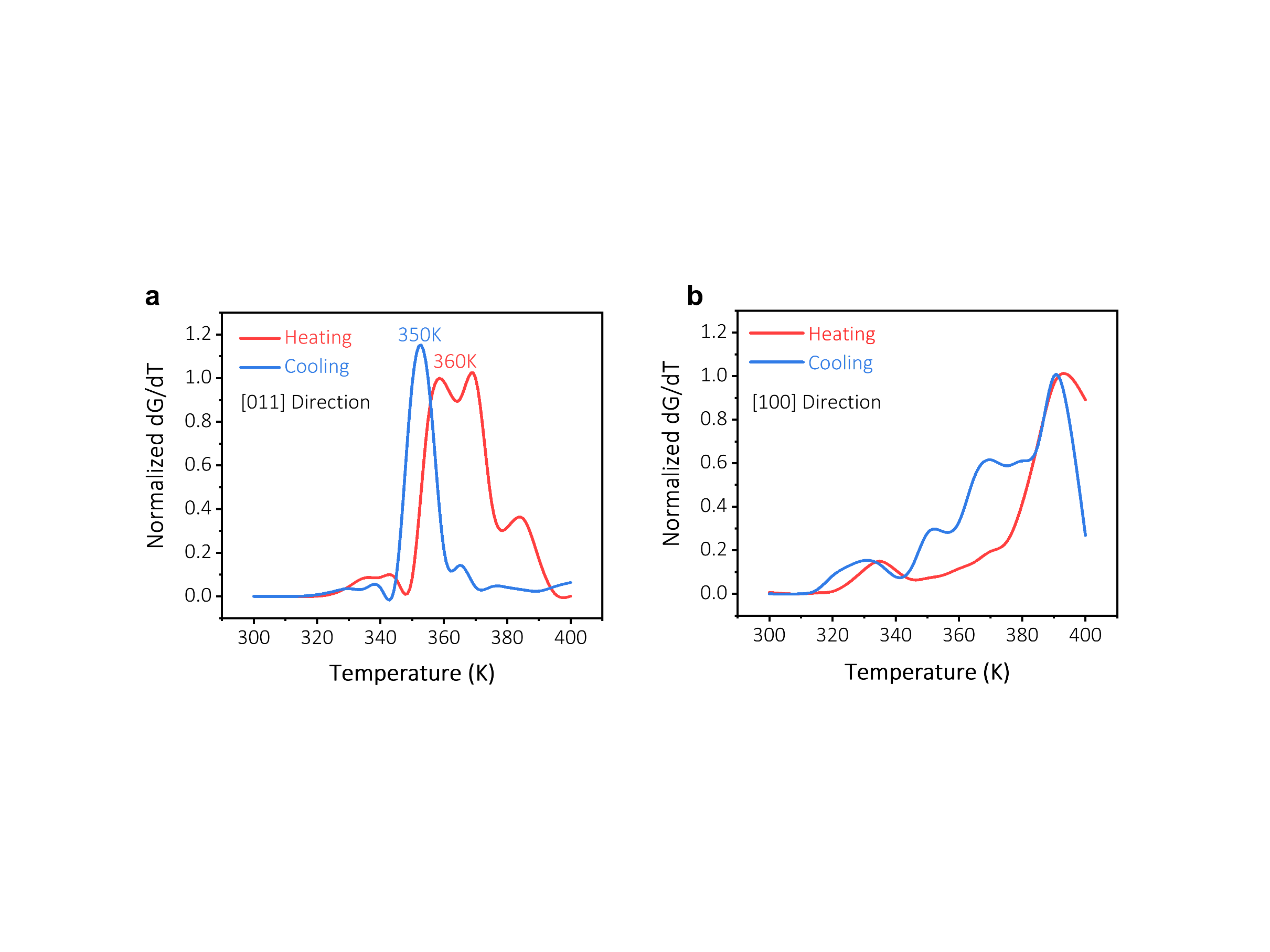


**Figure S22. Electrical properties of thin VO_2_ device.** a, b) The [011] and [100] direction derivative curves of conductance-temperature, dG/dT, for heating and cooling, respectively.

1. **The evolution of electrical anisotropic ratio in thick VO_2_ nanoflake**

For comparison, we have also done the same test on a thick VO_2_ nanoflake (**Fig. S23a-b**). Different from the thin VO_2_ nanoflake, the conductance-temperature curves in the thick sample displayed a distinctly sharp change (**Fig. S23c-d**), which can be attributed to the negligible stress in the thick sample. Previous studies revealed that phase transition temperature can be modulated by stress. Considering the distribution of stress in VO_2_ was not strictly uniform, the influence of stress on the transition temperature was therefore not uniform as well, resulting in a wide range distribution of the transition temperature and a gradual conductance change.

**
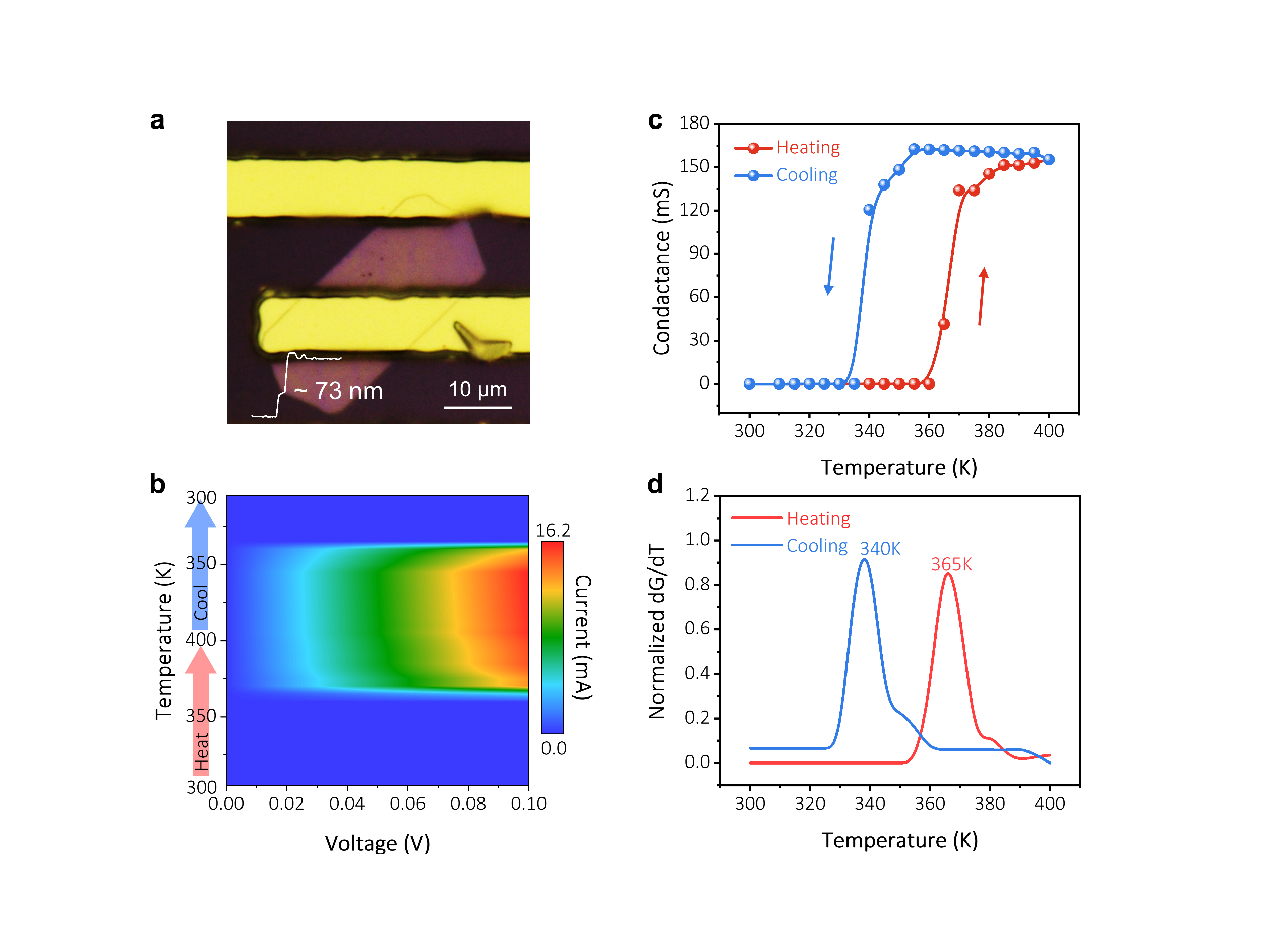
**

**Figure S23. Electric properties of the thick M1-VO_2_ device.** a) Optical image of the thick M1-VO_2_ device. b) Temperature-dependent anisotropic conductance ratio at varied voltages displayed in 2D mode. c) Conductance-temperature curves during the heating and cooling process extracted from (b) at a bias voltage of 0.1 V. d) The derivative curve of conductance-temperature, dG/dT, for the heating and cooling process.

**References**

1. Okimura K*, et al.* Temperature-dependent Raman and ultraviolet photoelectron spectroscopy studies on phase transition behavior of VO_2_ films with M1 and M2 phases. *J. Appl. Phys.* **115,** 153501 (2014).

2. Zhang S*, et al.* Direct correlation of structural domain formation with the metal insulator transition in a VO_2_ nanobeam. *Nano Lett.***9,** 4527-4532 (2009).

3. Wu, Y. F., et al. Spectroscopic analysis of phase constitution of high quality VO2 thin film prepared by facile sol-gel method. AIP Adv. **3**, 042132 (2013).

4. Jones, Andrew C., *et al.* Nano-optical investigations of the metal− insulator phase behavior of individual VO2 microcrystals. *Nano Lett.* **10**, 1574-1581 (2010).

5. Cao, J., *et al.* Extended mapping and exploration of the vanadium dioxide stress-temperature phase diagram. *Nano Lett.* **10,** 2667-2673 (2010).

6. Guo H*, et al.* Mechanics and dynamics of the strain-induced M1-M2 structural phase transition in individual VO_2_ nanowires. *Nano Lett.* **11,** 3207-3213 (2011).

7. Cao J*, et al.* Strain engineering and one-dimensional organization of metal-insulator domains in single-crystal vanadium dioxide beams. *Nat. Nanotechnol.* **4,** 732-737 (2009).

8. Park JH*, et al.* Measurement of a solid-state triple point at the metal-insulator transition in VO_2_. *Nature* **500,** 431-434 (2013).

9. Hidnert, Peter *et al*. Some physical properties of mica. *J. Res. Natl. Inst. Stand. Technol.* 35, 309-310 (1945).

10. Abaqus. V.6.14 Documentation. *Dassault Systemes Simulia Corporation* **651,** :6-2 (2014).

11. Huang Q*, et al.* Macroscopic simulation of membrane wrinkling for various loading cases. *Int. J. Solids Struct.* **64-65,** 246-258 (2015).

12. Nayyar V*, et al.* Stretch-induced stress patterns and wrinkles in hyperelastic thin sheets. *Int. J. Solids Struct.* **48,** 3471-3483 (2011).

13. M. Zhong, H. Meng, S. Liu, H. Yang, W. Shen, C. Hu, J. Yang, Z. Ren, B. Li, Y. Liu, J. He, Q. Xia, J. Li, Z. Wei, In-Plane Optical and Electrical Anisotropy of 2D Black Arsenic, *ACS Nano.* **15,** 1701–1709 (2021).

14. F. Xia, H. Wang, Y. Jia, Rediscovering black phosphorus as an anisotropic layered material for optoelectronics and electronics, *Nat. Commun.* **5,** 4458 (2014).

15. S.W. Lee, L. Qiu, J.C. Yoon, Y. Kim, D. Li, I. Oh, G.-H. Lee, J.-W. Yoo, H.-J. Shin, F. Ding, Z. Lee, Anisotropic Angstrom-Wide Conductive Channels in Black Phosphorus by Top-down Cu Intercalation, *Nano Lett.* **21,** 6336–6342 (2021).

16. L. Li, P. Gong, D. Sheng, S. Wang, W. Wang, X. Zhu, X. Shi, F. Wang, W. Han, S. Yang, K. Liu, H. Li, T. Zhai, Highly In‐Plane Anisotropic 2D GeAs _2_ for Polarization‐Sensitive Photodetection, *Adv. Mater.* **30,** 1804541 (2018).

17. L. Li, W. Wang, P. Gong, X. Zhu, B. Deng, X. Shi, G. Gao, H. Li, T. Zhai, 2D GeP: An Unexploited Low-Symmetry Semiconductor with Strong In-Plane Anisotropy, *Adv. Mater.* **30,** 1706771 (2018).

18. Y. Fang, F. Wang, R. Wang, T. Zhai, F. Huang, 2D NbOI_2_ : A Chiral Semiconductor with Highly In‐Plane Anisotropic Electrical and Optical Properties, *Adv. Mater.* **33,** 2101505 (2021).

19. Z. Shu, Q. Peng, P. Huang, Z. Xu, A.A. Suleiman, X. Zhang, X. Bai, X. Zhou, T. Zhai, Growth of Ultrathin Ternary Teallite (PbSnS_2_) Flakes for Highly Anisotropic Optoelectronics, *Matter.* **2,** 977–987 (2020).

20. L. Pi, C. Hu, W. Shen, L. Li, P. Luo, X. Hu, P. Chen, D. Li, Z. Li, X. Zhou, T. Zhai, Highly In‐Plane Anisotropic 2D PdSe_2_ for Polarized Photodetection with Orientation Selectivity, *Adv. Funct. Mater.* **31,** 2006774 (2021).

21. P. Li, J. Zhang, C. Zhu, W. Shen, C. Hu, W. Fu, L. Yan, L. Zhou, L. Zheng, H. Lei, Z. Liu, W. Zhao, P. Gao, P. Yu, G. Yang, *Penta* ‐PdPSe: A New 2D Pentagonal Material with Highly In‐Plane Optical, Electronic, and Optoelectronic Anisotropy, *Adv. Mater.* **33,** 2102541 (2021).

22. E. Liu, Y. Fu, Y. Wang, Y. Feng, H. Liu, X. Wan, W. Zhou, B. Wang, L. Shao, C.-H. Ho, Y.-S. Huang, Z. Cao, L. Wang, A. Li, J. Zeng, F. Song, X. Wang, Y. Shi, H. Yuan, H.Y. Hwang, Y. Cui, F. Miao, D. Xing, Integrated digital inverters based on two-dimensional anisotropic ReS2 field-effect transistors, *Nat. Commun.* **6,** 6991 (2015).

23. R. Wang, F. Zhou, L. Lv, S. Zhou, Y. Yu, F. Zhuge, H. Li, L. Gan, T. Zhai, Modulation of the Anisotropic Electronic Properties in ReS2 via Ferroelectric Film, *CCS Chem.* **1,** 268-277 (2019).

24. G. Ghimire, K.P. Dhakal, W. Choi, Y.A. Esthete, S.J. Kim, T.T. Tran, H. Lee, H. Yang, D.L. Duong, Y.-M. Kim, J. Kim, Doping-Mediated Lattice Engineering of Monolayer ReS_2_ for Modulating In-Plane Anisotropy of Optical and Transport Properties, *ACS Nano.* **15,** 13770–13780 (2021).

25. G. Kim, B. Feng, S. Ryu, H.J. Cho, H. Jeen, Y. Ikuhara, H. Ohta, Anisotropic Electrical Conductivity of Oxygen-Deficient Tungsten Oxide Films with Epitaxially Stabilized 1D Atomic Defect Tunnels, *ACS Appl. Mater. Interfaces.* **13,** 6864–6869 (2021).

26. Y. Wang, S. Yao, P. Liao, S. Jin, Q. Wang, M.J. Kim, G.J. Cheng, W. Wu, Strain‐Engineered Anisotropic Optical and Electrical Properties in 2D Chiral‐Chain Tellurium, *Adv. Mater.* **32,** 2002342 (2020).

27. S. Deng, E. Gao, Y. Wang, S. Sen, S.T. Sreenivasan, S. Behura, P. Král, Z. Xu, V. Berry, Confined, Oriented, and Electrically Anisotropic Graphene Wrinkles on Bacteria, ACS Nano. 10, 8403–8412 (2016).
